# Thermoelectric properties plus phonon and de Haas–van Alphen frequencies of hole/electron-doped $\hbox {CeIn}_3$

**DOI:** 10.1038/s41598-021-04058-1

**Published:** 2022-01-13

**Authors:** M. Yazdani-Kachoei, S. Rahimi, R. Ebrahimi-Jaberi, J. Nematollahi, S. Jalali-Asadabadi

**Affiliations:** Department of Physics, Faculty of Physics, University of Isfahan (UI), Hezar Jerib Avenue, Isfahan, 81746-73441 Iran

**Keywords:** Condensed-matter physics, Theory and computation

## Abstract

We investigate temperature, pressure, and localization dependence of thermoelectric properties, phonon and de Haas–van Alphen (dHvA) frequencies of the anti-ferromagnetic (AFM) CeIn$_3$ using density functional theory (DFT) and local, hybrid, and band correlated functionals. It is found that the maximum values of thermopower, power factor, and electronic figure of merit of this compound occur at low (high) temperatures provided that the 4f-Ce electrons are (not) localized enough. The maximum values of the thermopower, power factor, electronic figure of merit (conductivity parameters), and their related doping levels (do not) considerably depend on the localization degree and pressure. The effects of pressure on these parameters substantially depend on the degree of localization. The phonon frequencies are calculated to be real which shows that the crystal is dynamically stable. From the phonon band structure, the thermal conductivity is predicted to be homogeneous. This prediction is found consistent with the thermal conductivity components calculated along three Cartesian directions. In analogous to the thermoelectric properties, it is found that the dHvA frequencies also depend on both pressure and localization degree. To ensure that the phase transition at Néel temperature cannot remarkably affect the results, we verify the density of states (DOS) of the compound at the paramagnetic phase constructing a non-collinear magnetic structure where the angles of the spins are determined so that the resultant magnetic moment vanishes. The non-collinear results reveal that the DOS and whence the thermoelectric properties of the compound are not changed considerably by the phase transition. To validate the accuracy of the results, the total and partial DOSs are recalculated using DFT plus dynamical mean-field theory (DFT+DMFT). The DFT+DMFT DOSs, in agreement with the hybrid DOSs, predict the Kondo effect in this compound.

## Introduction

Cooling is a crucial requirement for many electronic and optoelectronic devices such as integrated circuit devices or semiconductor lasers. In fact, lower temperatures can significantly increase the efficiency of these devices. However, the common cooling mechanisms are not always useful specifically in small scale applications. Therefore, searching for new cooling methods is an active topic for vast areas of science and engineering^1–8^. Peltier cooling mechanism using thermoelectric (TE) materials is a good candidate instead of common refrigeration methods. This mechanism is more useful in small scale applications. Recent studies reveal the importance and applications of this mechanism^9–13^. Some heavy Fermion compounds have been proposed as candidates for thermoelectric cooling devices^14–16^. However, the low efficiency of the available TE materials limits their applications in practice for scientists and engineers. This is the most problem in applications of the Peltier cooling mechanism for engineering the materials. Thus, tremendous theoretical and experimental efforts have been invested to find new TE materials with high efficiency or increase efficiency of the available TE materials^17–20^. Therefore in this work, we concentrate on the efficiency of one of these TE materials as discussed through the paper in details. The thermoelectric efficiency of a material is characterized by its figure of merit ($ZT$) parameter, where $Z$ is $\frac{\sigma S^{2}}{\kappa }$, $\sigma$ is electrical conductivity, $S$ is the Seebeck coefficient or thermopower, and $\kappa$ is the thermal conductivity of the compound. Therefore, higher thermopower and electrical conductivity accompanied by lower thermal conductivity leads to higher thermoelectric efficiency. It has been reported in literatures^21–26^ that Ce-based compounds have high thermopower and electrical conductivity at low temperatures. Therefore, Ce-based compounds can be good candidates for thermoelectric cooling. CeIn$_3$ is one of these materials that exhibits good thermoelectric properties and great Z value, especially at low temperatures (T $\ll$ 300 K)^27,28^. This material crystalizes in the $Pm\bar{3}m$ space group with Néel temperature of T$_\text {N}$ = 10 K^29^. An important point which should be considered is that in the thermoelectric applications the movement is toward cheap, abundant, environmentally friendly materials. Ce is the most abundant rare-earth material^30–32^. Indium is almost as abundant as silver, but it is much easier to recover indium, since it can be typically found in zinc, iron, lead and copper ores^33,34^. This can be potentially an important advantage for the applications of CeIn$_3$ in the thermoelectric cooling devices. Experimental results have shown that the electron or hole doping can affect the thermoelectric as well as structural, magnetic and electrical properties of CeIn$_3$^35–38^. The electrical conductivity of this compound can be increased by electron doping^36,39,40^, which ultimately can affect the TE efficiency of the material. Up to now, very extensive theoretical and experimental studies have been performed to shed light into the electronic and magnetic properties of CeIn$_3$^35,36,39,41–46^ . Recently, we also discussed^47^ the effects of pressure and degree of localization of the 4f-Ce electrons on the structural and magnetic properties as well as the electronic structure of CeIn$_3$. Our calculated total magnetic moments of CeIn$_3$ per cerium atom at various pressures using a variety of exchange-correlation functionals (XCFs) along with available experimental data were reported in the Table 2 of our previous work^47^. We using the B3PW91 XCF with $\alpha =0.20$ predicted the value of the $0.67~ \mu _B$ for the magnetic moment of this compound at zero pressure which was found in good agreement with the experimental result, i.e., $0.65 ~\mu _B$^48^.

However, investigation of its thermoelectric efficiency has been restricted to some experimental measurements^27,28,49^, and a comprehensive theoretical analysis has been lacking for this compound, despite its high thermoelectric efficiency. Therefore, in this work in continue of our experiences on this particular unexpected case^43,47,50–55^, we theoretically demonstrate the thermoelectric properties and thermoelectric efficiency of hole/electron doped CeIn$_3$ within the density functional theory (DFT)^56,57^ and Boltzmann theory^58,59^. Like electron/hole doping, hydrostatic pressure can also affect TE properties. Thus, we investigate the effects of pressure on the TE properties of CeIn$_3$, as well. Furthermore, the pressure dependency of the degree of Ce 4f-electrons localization has been demonstrated through the de Haas–van Alphen (dHvA) experiments for this compound^44,45^. This motivated us to investigate the effects of the localization degree of Ce 4f-electrons on the TE properties of CeIn$_3$ too. In this regard, we employ the hybrid functional B3PW91 with various $\alpha$ parameters as well as the LDA approaches to control the degree of localization and model the exchange-correlation energy in the electronic structure calculations. The thermoelectric calculations are performed using three relaxation time ($\tau$) models; (*i*) a constant $\tau$ model, (*ii*) an energy dependent $\tau$ model with lower scattering parameter^60^, and (*ii*) an energy dependent $\tau$ model with higher scattering parameter^60^, as discussed in the next section. In addition to the collinear AFM phase, we have also studied the electronic structure of the system at paramagnetic (PM) phase using a non-collinear magnetism scheme. These calculations reveal negligible differences between the calculated AFM and PM densities of states (DOSs) for CeIn$_3$. Furthermore, we have studied the Kondo effects in this compound using density functional theory (DFT) plus dynamical mean field theory (DFT+DMFT). Our DFT+DMFT results predict the Kondo effect in this compound. The dHvA and phonon calculations are performed in this article. The phonon calculations reveal the high degree of localization for phonon states in this compound.

## Calculation details

**Figure 1:**
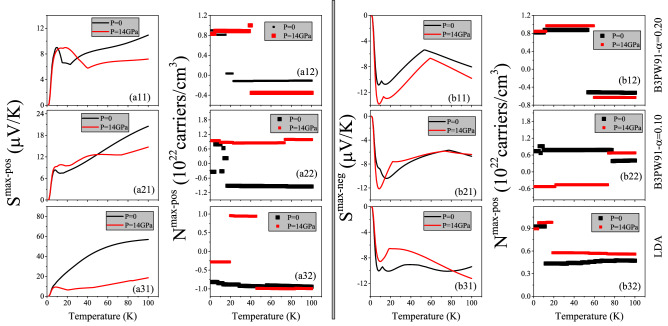
(**ai1**) Maximum positive or hole-like of spin up Seebeck coefficient ($S^{max-pos}$) and (**bi1**) maximum negative or electron-like of spin up Seebeck coefficient ($S^{max-neg}$) of hole/electron doped CeIn$_3$ versus temperature calculated by B3PW91 with $\alpha$  = 0.20, 0.10 and LDA for *P* = 0 and 14 GPa pressures. Doping levels related to $S^{max-pos}$ and $S^{max-neg}$ are displayed in the (**ai2**) and (**bi2**) panels, respectively. The i-index varies from 1 to 3. In the AFM phase, spin down results are the same as spin up results.

The electronic calculations in the present work are performed within the density functional theory (DFT)^56,57^ using the full potential APW+lo method^61^ as implemented in the WIEN2k code^62^ in the presence of spin-orbit coupling (SOC) and spin polarization. Our calculations are performed using two different lattice parameters 4.68 Å  calculated at* P* = 0 and 4.44 Å  calculated at *P* = 14 GPa by B3PW91-$\alpha =0.20$^47^. As discussed in our previous study^47^ using the results presented in Table 1 and Fig. 1 of the latter reference and taking both of the lattice parameter and bulk modulus as well as their fluctuations over the $\alpha$ parameter of the hybrid functional and Hubbard U parameter of the GGA+U functional into account, overall the structural properties predicted by B3PW91 with $\alpha =0.20$ are more consistent with the experimental results compared to those of the other considered exchange-correlation functionals (XCFs). As we aim to study the considered case in the antiferromagnetic (AFM) phase, we construct a $2\times 2\times 2$ supercell with two Ce atoms; one with spin up and another one with spin down. The grids of $12\times 12\times 12$ in the scheme of Monkhorst-Pack^63^ are selected for the mesh of k-points within the electronic structure calculations. The cutoff parameters $K_{max}$, $l_{max}$, and $G_{max}$ are set to $7R_{MT}^{-1}$, 10, and 16 Bohr$^{-1}$, respectively. The muffin-tin radii ($R_\text {MT}$) are chosen to be 2.2 a.u. for In and 2.8 a.u. for Ce. The GGA+U with $\text {U}_{\text {eff}}=5.5~\text {eV}$, B3PW91^64,65^ with $\alpha =$0.10 and 0.20 as well as the LDA functionals are used to describe the exchange-correlation term. In the hybrid functionals generally some portion of the exchange term in the semilocal functional is replaced by the Hartree-Fock (HF) exchange so that general form of these functionals can be written as:

1 $$\begin{aligned} E^{hybrid}_{xc}=E^{SL}_{xc}+ \alpha (E^{HF}_{x}-E^{SL}_{x}), \end{aligned}$$

where $\alpha$ is a parameter which determines the portion of the Hartree-Fock exchange, $E^{hybrid}_{xc}$ and $E^{SL}_{xc}$ are the hybrid and semilocal exchange-correlation functionals, as well as $E^{HF}_{x}$ and $E^{SL}_{x}$ are Hartree-Fock and semilocal exchange functionals, respectively. The B3PW91 hybrid functional (which we have used in this work) is:

2 $$\begin{aligned} {\begin{matrix} E^{B3PW91}_{xc}= (1-\alpha )E^{LSDA}_{xc}+ \alpha E^{HF}_{x}+ \beta \Delta E^{B88}_{x} +E^{LSDA}_{c}+ \gamma \Delta E^{PW91}_{c}, \end{matrix}} \end{aligned}$$

where $\Delta E^{B88}_{x}$ is the Becke’s 1988 gradient correction to the exchange functional^66^, and $\Delta E^{PW91}_{c}$ is the Perdew-Wang gradient correction for the correlation functional^67^.

The quality of physical properties predicted theoretically may depend on the functionals used in the DFT calculations and vary from case to case. It may also depend on the methods used and the pressures applied. All the properties of the compound under study cannot be always reproduced in excellent agreement with the experimental data by a single functional only, as discussed in Ref.^47^. For instance, in one side, the experimental lattice constant of CeIn$_3$^36^ can be well reproduced by PBE-GGA functional, whereas the latter functional fails to predict the magnetic moment of the compound^48^. On the other hand, the B3PW91 functional with $\alpha =0.2$ predicts the magnetic moment of CeIn$_3$ consistent with experiment^48^ but this hybrid functional with $\alpha =0.2$, compared to PBE-GGA, cannot satisfactorily predict the lattice constant of the system. Moreover, although the WC-GGA can predict the bulk modulus of CeIn$_3$ better than the other considered XCFs compared to the experiment^37^, some of the other quantities than bulk modulus can be more satisfactorily predicted by the other considered XCFs than WC-GGA. Furthermore, according to our previous results^47^ because of the pressure dependency of the degree of localization of the 4f-Ce electrons it is more appropriate to use different functionals at different pressure regimes, i.e., functionals with high (low) degree of localization would be used at very low (high) pressures. For instance, in this study, we see that the dHvA frequency can be better predicted by LDA at high pressures which is consistent with the low localization degree originating from the high pressures imposed. These evidences show that the available methods and functionals are not still so general that can be reliably used for any physical quantities and cases. Therefore, in this work, we have used various functionals and/or methods rather than a specific functional and/or method.

The transport properties are calculated by the BoltzTraP code^68^ which is based on the Boltzmann theory^58,59^. Since the reliability of the thermoelectric properties depend on the density of the k-mesh, a dense mesh of 100000 k-points is considered for the thermoelectric calculations. Using our program, as written in pages 7–10 of the “*Supplementary Information*” provided for Ref. ^69^, we calculate the maximum values of hole-like or positive Seebeck coefficient ($S^{max-pos}$), the maximum values of electron-like or negative Seebeck coefficient ($S^{max-neg}$), the maximum values of electrical conductivity per relaxation time ($\sigma ^{max}\tau$), the maximum values of electronic part of thermal conductivity per relaxation time ($\kappa ^{max}\tau$), the maximum values of power factor per relaxation time ($PF^{max}\tau$), and the maximum values of electronic figure of merit ($Z_e^{max}$) versus temperature up to T = 100 K by step 1 K. For more details, see also Fig. 6 of the “*Supplementary Information*” of the Ref. ^69^, where the flowchart of the program is shown. We also calculate the doping levels that lead to these maximum values. To this end, the program looks for the highest values of the thermoelectric parameters and their related doping levels which are predicted at each temperature by BoltzTraP code. Then, the temperature is increased by step 1 K and the search is repeated up to the end of temperature range ($T_{max}$). For Seebeck coefficient the minimum values are also searched for finding $S^{max-neg}$. We have limited the doping level between − 10$^{21}$ and 10$^{21}$ carriers/cm$^3$ (equivalent to − 1.6 and 1.6 carries/uc) to be realized in experiments. This doping level range now also well agrees with the previous studies on the other cases^70^. In addition to the constant $\tau$ model, we have applied the $\tau =\tau _0 \epsilon ^{(r-1/2)}$ model^60^ for relaxation time, as implemented in the BoltzTraP code, using B3PW91-$\alpha =0.20$ at *P* = 0. In this model, the $\tau _0$ is the reference life time (in femto second), *r* is the scattering parameter, and $\epsilon =(hk)^2/2m^\star )$^60^, where *k* is the wave vector and $m^\star$ is the effective mass. We consider $\tau _0=5$ and two values for *r* in the calculations; $r=2$ & 4. We call the $\tau =\tau _0 \epsilon ^{(r-1/2)}$ with $r=2 (r=4)$ Model1 (Model2) in this paper.

As discussed in the previous section, the case under study transforms to paramagnetic (PM) phase above the T = 10 K. Therefore, we have performed noncollinear magnetism calculations to simulate the PM phase at temperatures higher than 10 K. However, this cannot be done by the standard WIEN2k code. By the standard WIEN2k code collinear magnetic phases such as FM and AFM can be studied. But, PM phase is a noncollinear magnetic phase which requires more special treatments. Fortunately, the PM phase can be studied by the noncollinear magnetism version of the WIEN2k code which is WIENncm code^71^. The self-consistent field convergence by WIENncm is more difficult and larger computational resources are required. Despite these difficulties, we have performed the PM calculations using the non-collinear magnetism version of the WIEN2k code, called WIENncm code^71^. These calculations have been done using a $2 \times 2 \times 1$ supercell, including four Ce atoms within the GGA+U approach with $\text {U}_{\text {eff}}=5.5~\text {eV}$ at zero pressure. In the WIENncm code^71^ the directions of spins are determined by the polar angels ($\phi , \theta$).

It is worth mentioning that, in one side, a large supercell, including many atoms with complicated spin configuration, is required for simulating the PM phase with high accuracy. The self-consistent-convergence of the non-collinear magnetism is slower and more difficult than that of the collinear magnetism and whence larger computational resources are demanded for simulating the PM phase. The non-collinear calculation performed by WIENncm code is more time consuming than that of the standard WIEN2k code. Thus, here, in anticipation of further investigations, we limited our constructed supercell to a static non-collinear structure containing 4 magnetic atoms. Such a limited treatment can hardly be called PM phase. Consequently, this small non-collinear structure may be considered only as a first-order approximation beyond the collinear structure towards the non-collinear PM phase. On the other hand, the localized magnetic moments and their very weak interaction (as reflected by low Néel temperature) indicate that an AFM structure could be a reasonable model for many properties of the PM phase without any non-collinear calculations. Furthermore, the current version of the WIENncm code does not support the hybrid XCFs and whence it does not work with the B3PW91 XCF. Thus, despite the success of the hybrid B3PW91 XCF with $\alpha =0.20$, as reported in Ref. ^47^, it is currently impossible to calculate the PM phase using WIENncm and B3PW91 XCF with $\alpha =0.20$, due to the above practical restriction. Therefore, taking all the above points into account, most of the calculations are performed by the hybrid XCF within the standard WIEN2k code for the collinear AFM phase, whereas the non-collinear PM calculations are performed by the GGA+U within the WIENncm code.

The dHvA frequencies are calculated using the supercell k-space extremal area finder (SKEAF) code^72^. By this code, we have extracted the extremal electron/hole Fermi surface orbits for a set of band energies generated by the DFT-based WIEN2k package. To this end, we have started from the set of calculated band energies, and then constructed a large interpolated k-space supercell which is broken into slices perpendicular to the desired magnetic field direction by means of the SKEAF program and used this to determine dHvA frequencies.

We have used the PHONOPY code^73^ to calculate the phonon frequencies. In the PHONOPY^73^ force constants are generated based on finite displacement method and harmonic approximation. These calculations are done using a $2\times 2\times 2$ supercell.

The DMFT calculations are performed using the embedded DMFT (eDMFT) code^74^ as a more accurate code to calculate the total DOS and partial $4f_{5/2}$ and $4f_{7/2}$ DOSs of our compound at *P* = 0. By the eDMFT code, we have combined our DFT calculations with the dynamical mean field theory (DMFT). To this end, the single-particle Green’s function is expanded in terms of the FP-LAPW bases, and then the single-particle Green’s function as well as the electronic charge are self-consistently extracted.

In summary, here, we have used the following codes for the summarized corresponding purposes: (*i*) WIEN2k^62^, as the backbone of our full-potential APW+lo^61^ DFT^56,57^ study, to calculate the electronic structures and X-Ray spectrum for the AFM phase of the system in question and generate the necessary outputs for the other codes, (*ii*) BoltzTrap^68^ to calculate the thermoelectric properties using the relevant outputs generated by the WIEN2k code and our post-processing program Ref. ^69^, (*iii*) SKEAF^72^ to calculate the dHvA frequencies using the relevant outputs generated by the WIEN2k code, (*iv*) PHONOPY^73^ to calculate the phonon frequencies and dynamic stability of the system using the relevant outputs generated by the WIEN2k code, (*v*) WIEN2k plus eDMFT^74^ to perform DFT+DMFT calculations to obtain total and partial DOSs, and (*vi*) WIENncm^71^ to perform non-collinear magnetism calculations to simulate the PM phase of the system. In this study, several functionals are also used, including LDA, PBE-GGA, GGA + U with $\text {U}_{\text {eff}}=5.5~\text {eV}$, B3PW91 with $\alpha = 0.10$ and $\alpha = 0.20$, to consider different degrees of localization at* P* = 0 and* P* = 14 GPa. When it is practically possible, the latter functionals are used because the localization degree can vary by pressure and whence it can affect the properties predicted. Since the hybrid functional is not currently implemented in the WIENncm code, however, the PM phase is studied by GGA + U only.

## de Haas–van Alphen (dHvA) frequencies

**Table 1:** dHvA frequencies of CeIn$_3$ Fermi surface branches at zero and 14 GPa pressures calculated by the B3PW91-$\alpha =0.20$, B3PW91-$\alpha =0.10$ and LDA XCFs.

Band	XCF					
	B3PW91-$\alpha =0.20$		B3PW91-$\alpha =0.10$		LDA	
	*P* = 0	*P* = 14 GPa	*P* = 0	*P* = 14 GPa	*P* = 0	*P* = 14 GPa
$\gamma 1$	0.3	0.1	0.1			
			0.2			
$\gamma 2$	0.1	0.2	0.2	0.4	1.2	0.2
	0.4	0.4	0.5		1.3	1.8
	0.8	0.6			3.0	3.5
	1.0	0.9				5.1
	1.5	1.0				5.9
	1.8	1.8				6.9
	2.0	1.9				9.3
	2.2					
	9.1					
$\gamma 3$	0.1	0.1	0.2	0.2	0.9	0.1
	0.2	0.2	0.3	0.4	1.9	0.7
	1.6	0.6	0.4	0.5	4.8	0.8
	2.4	2.0	1.2	0.7	6.2	1.0
	2.9	3.0	2.5	1.6		2.0
		3.9	3.1	3.0		2.6
		4.3	4.5	4.0		4.5
			6.9	4.3		6.6
				4.5		
				5.0		
				5.3		
				5.9		
				6.3		

The unit of reported frequencies is kilo Tesla (kT).

In this section, we discuss the de Haas–van Alphen (dHvA) frequencies related to the branches of Fermi surfaces of CeIn$_3$ at *P* = 0 and 14 GPa using the three considered XCFs. To this end, first, let us consider the electronic structures of the system. The band structure and Fermi surfaces of CeIn$_3$ were discussed in our previous work^47^ at zero and 14 GPa pressures using B3PW91-$\alpha =0.20$, B3PW91-$\alpha =0.10$ and LDA XCFs. Fig. 4 of Ref. ^47^ exhibited the band structures of CeIn$_3$ at two pressures and three XCFs. As shown in this figure, three bands called $\gamma _1, \gamma _2, \gamma _3$ crossed the Fermi level in CeIn$_3$ band structure using B3PW91-$\alpha =0.20$ at the two considered pressures (at *P* = 0 and 14 GPa). The same result was observed using B3PW91-$\alpha =0.10$ at zero pressure. But, B3PW91-$\alpha =0.10$ XCF predicted that the $\gamma _1$ band was located below the Fermi level at *P* = 14 GPa. Thus, only two bands ($\gamma _2, \gamma _3$) crossed the Fermi level using B3PW91-$\alpha =0.10$ at *P* = 14 GPa. The LDA XCF predicted that only two bands ($\gamma _2, \gamma _3$) could cross the Fermi level at both zero and 14 GPa pressures. Table 3 of Ref. ^47^ show the characters, including the maximum energies (E$_{max}$), minimum energies (E$_{min}$), band widths, and occupation numbers of bands crossing the Fermi level in the CeIn$_3$ band structure, at *P* = 0 and 14 GPa using the three considered XCFs. The Fermi surface branches of these bands were also presented in Fig. 5 of Ref. ^47^.

The dHvA frequencies in kilo Tesla (kT) are presented in Table 1. R. Settai et al. experimentally measured^45^ the dHvA to be 3.22 kT for the case under study at zero pressure. In agreement with the latter experimental value^45^, our dHvA calculations performed at zero pressure using B3PW91-$\alpha =0.20$, B3PW91-$\alpha =0.10$, and LDA XCFs predict the values of 2.9, 3.1, and 3.0 kT, respectively. Moreover, in this experimental work^45^ it was also found that imposing pressures higher than 2.7 GPa on CeIn$_3$ caused the appearance of a new branch with 9.89 kT dHvA frequency, due to the transition of the 4f Ce electrons from high localized to itinerant. The latter point is consistent with the fact that the degree of localization decreases as pressure increases, as discussed in Ref. ^47^. This implies that the LDA with low degree of 4f electron localization can be more suitable at higher pressures, see Ref. ^47^. Therefore, we have also considered the LDA functional for the dHvA calculations at the high pressure. Our calculations performed at *P* = 14 GPa using LDA XCF, having low degree of 4f electron localization, predict the value of 9.3 kT which is consistent with the above experimental value^45^. Our results also show that the dHvA frequency related to the $\gamma _1$ band is decreased by imposing pressure using B3PW91-$\alpha =0.20$ XCF. Reducing the $\alpha$ parameter leads to the same trend at *P* = 0; the dHvA frequency related to the $\gamma _1$ band calculated by B3PW91-$\alpha =0.10$ is slightly smaller than that calculated by B3PW91-$\alpha =0.20$. No frequency is reported for $\gamma _1$ band using B3PW91-$\alpha =0.10$ XCF at *P* = 14 GPa, because this band does not contribute in the Fermi surface using this XCF at *P* = 14 GPa. The same result is obtained using LDA at *P* = 0 & 14 GPa. A high frequency (about 9 kT) is predicted by B3PW91-$\alpha =0.20$ for the $\gamma _2$ band at *P* = 0. But, this high frequency is not observed by B3PW91-$\alpha =0.10$ at *P* = 0 & 14 GPa, as well as by LDA at *P* = 0. A frequency about 6 kT at both *P* = 0 & 14 GPa is predicted by B3PW91-$\alpha =0.10$, which is not seen when B3PW91-$\alpha =0.20$ is applied.

## Thermoelectric properties

The Seebeck coefficient, electrical conductivity, and electronic part of the thermal conductivity tensors are calculated for the CeIn$_3$ using the following formulas^68,75^, respectively:

3 $$\begin{aligned}&S_{\alpha \beta }(\mu , T)= \frac{1}{eT\Omega \sigma _{\alpha \beta }(\mu , T)}\int \sigma _{\alpha \beta }(\varepsilon )(\varepsilon -\mu ) \left[ -\frac{\partial f_0(\mu , \varepsilon , T)}{\partial \varepsilon }\right] d\varepsilon , \end{aligned}$$

4 $$\begin{aligned}&\sigma _{\alpha \beta }(\mu , T) = \frac{1}{\Omega }\int \sigma _{\alpha \beta }(\varepsilon )\left[ -\frac{\partial f_0(\mu , \varepsilon , T)}{\partial \varepsilon }\right] d\varepsilon , \end{aligned}$$

5 $$\begin{aligned}&\kappa _{\alpha \beta }(\mu , T)= \frac{1}{e^2T\Omega }\int \sigma _{\alpha \beta }(\varepsilon )(\varepsilon -\mu )^2 \left[ -\frac{\partial f_0(\mu , \varepsilon , T)}{\partial \varepsilon }\right] d\varepsilon , \end{aligned}$$

where $\mu$ is the carrier concentration, $\Omega$ is the volume of unit cell, $\alpha$ and $\beta$ are the tensor indices, $f _0$ is the Fermi-Dirac distribution function, $e$ is the carrier charge, and

6 $$\begin{aligned} \sigma _{\alpha \beta }(\varepsilon )= \frac{1}{N}\sum _{i,k}\sigma _{\alpha \beta }(i,k)\frac{\delta (\varepsilon -\varepsilon _{i,k})}{\delta \varepsilon }, \end{aligned}$$

where

7 $$\begin{aligned} \sigma _{\alpha \beta }(i,k)=e^2\tau _{i,k}\upsilon _{\alpha }(i,k)\upsilon _{\beta }(i,k). \end{aligned}$$

Here, N is the number of k points, $\tau$ is the relaxation time, and $\upsilon _{\alpha }(i,k)$ is the $\alpha -$component of the group velocity. It is noticeable that the thermal and electrical conductivities are calculated with respect to the relaxation time, here. As would be seen from Eqs. 4 to 5, the thermoelectric parameters are functions of $\mu$ and T. Hence, there are several values for thermoelectric parameters at every temperature. Technical setup is discussed in “Calculation details” section. Ultimately power factor is also calculated as $ZT = \frac{\sigma S^2}{\kappa }$ using Eqs. 3,  4, and 5. Let us subsequently discuss the Seebeck coefficient, electrical conductivity, and thermal conductivity, as well as power factor separately in “Seebeck coefficient”, “Electrical conductivity”, and “Thermal conductivity” sections, as well as “Power factor” section, respectively.

### Seebeck coefficient

**Table 2:** The maximum values of positive (hole-like) and negative (electron-like) Seebeck coefficient of CeIn$_3$ at *P* = 0 and 14 GPa using B3PW91 with $\alpha$ = 0.20, 0.10 and LDA at specified temperatures T = 10, 50, and 100 K.

T(K)	B3PW91-$\alpha$ = 0.20		B3PW91-$\alpha$ = 0.10		LDA	
	*P* = 0	*P* = 14 GPa	*P* = 0	*P* = 14 GPa	*P* = 0	*P* = 14 GPa
***S*** ^***max−pos***^						
10	8.98	8.60	7.86	9.32	14.92	9.14
50	8.61	6.37	12.73	12.66	45.6	9.57
100	10.94	7.20	20.54	14.74	56.98	18.60
***S*** ^***max−neg***^						
10	− 10.05	− 13.62	− 9.01	− 11.97	− 9.82	− 8.57
50	− 5.74	− 7.97	− 6.88	− 6.60	− 9.22	− 7.62
100	− 8.02	− 9.81	− 6.70	− 6.40	− 9.38	− 11.21

The unit of numbers is $\frac{\mu V}{K}$.

As can be seen from formula $ZT=\frac{\sigma S^2}{\kappa }$, higher thermopower yields higher thermoelectric efficiency. This simple formalism can be used and applied by various ways such as electron/hole doping or imposing pressure. Here, we aim to find the maximum thermopower of electron/hole doped CeIn$_3$ compound at low temperatures, i.e. T $\le$ 100 K, and electron/hole doping around 10$^{21}$ carriers/cm$^3$. As comes from Eq. 3, Seebeck coefficient is a tensor with nine components. However, our calculations show that the diagonal elements of this tensor are very higher than the off-diagonal elements so that the off-diagonal components can be neglected. Furthermore, the cubic structure of CeIn$_3$ implies that all the diagonal elements of the Seebeck coefficient tensor are equal to each other. Thus, we only investigate the *xx*-component of the Seebeck coefficient, here. The maximum values of the hole-like (positive) [electron-like (negative)] spin up Seebeck coefficient of electron/hole doped of CeIn$_3$, i.e., $S^{max-pos}$ [$S^{max-neg}$], calculated using the B3PW91 XCF with $\alpha =$ 0.20, and 0.10 as well as LDA XCF are presented in the (ai1) [(bi1)] panels of Fig. 1 for i = 1–3. In the AFM phase spin up results are the same as spin down results. Therefore, we only report spin up results for our AFM case in this work. This figure displays the results at two different pressures *P* = 0 and *P* = 14 GPa. The values of $S^{max-pos}$ and $S^{max-neg}$ at the specified temperatures of T = 10, 50, 100 K are also tabulated in Table 2. The doping levels related to the maximum values of the hole-like and electron-like thermopowers are also displayed in (ai2) and (bi2) panels of the latter figure, respectively. We start our discussion with the results obtained by B3PW91-$\alpha$ = 0.20, as shown in the (a1j) and (b1j) panels of Fig. 1 for j = 1, 2. As presented in Fig. 1a11, the $S^{max-pos}$ shows a peak at very low temperatures around the T $\simeq$ 10 K. Kletowski^27^ experimentally observed such a behavior for CeIn$_3$^27^, reporting a peak with the value of 27 $\mu V/K$ for the Seebeck coefficient of CeIn$_3$ at zero pressure around T $\simeq$ 50 K. Figure 1a12 shows that this peak is due to the hole doping. After this peak, the $S^{max-pos}$ decreases as temperature increases up to about T $\simeq$ 23 K. But, for temperatures higher than T $\simeq$ 23 K, the $S^{max-pos}$ increases as temperature increases. Figure 1b11 predicts a negative peak for the $S^{max-neg}$ at low temperatures T $\simeq$ 15 K and *P* = 0. The value of this negative peak at low temperatures is about one and a half times higher than the value of the positive peak in the $S^{max-pos}$. Our results show that the hole doping leads to this negative peak in the $S^{max-neg}$, the same as the positive peak in $S^{max-pos}$. After the T $\simeq$ 15 K, the $S^{max-neg}$ is increased by increasing temperature up to about 60 K at *P* = 0, then it again decreases as temperature rises up to T = 100 K. Figure 1a21 and b12 indicate that in the most of the considered temperature range very low values of electron doping levels lead to both $S^{max-pos}$ and $S^{max-neg}$ at *P* = 0.

According to the experimental results^23,76,77^ the thermopower can be affected by imposing hydrostatic pressure. Therefore, we investigate the $S^{max-pos}$ and $S^{max-neg}$ at *P* = 14 GPa, as well. Our results show that applying pressure makes the $S^{max-pos}$ worse at most of the considered temperature range within the B3PW91 XCF with $\alpha$ = 0.20, and only improves it at very narrow temperature range. Applying pressure also shifts the peak of $S^{max-pos}$ from T$\simeq$ 10 K toward the higher temperatures using the B3PW91-$\alpha$ = 0.20, but does not change its value drastically. Fig. 1a12 reveals that the doping levels related to the $S^{max-pos}$ are also affected by imposing pressure at most of the considered temperature range; see Fig. 1a12 specifically from 15 to 40 K. In contrast to the $S^{max-pos}$, the $S^{max-neg}$ values are improved by imposing pressure at all of the considered temperature range within the B3PW91 XCF with $\alpha$ = 0.20. The $S^{max-neg}$ is decreased by imposing pressure within the B3PW91 XCF with $\alpha$ = 0.20, as displayed in Fig. 1b11. According to our calculations, the values of the electron/hole doping levels related to the $S^{max-neg}$ are increased by imposing pressure.

The dHvA experiments showed that the character of 4f-electrons is changed from localized to itinerant by applying pressure^44,45^. Furthermore, as we discussed in our recent work^47^, in agreement with the previous work^78^, the exchange-correlation energy of Ce-based compounds could not be satisfactorily described only by a single functional for every pressure. Therefore, for a specific pressure range an appropriate functional must be selected; band-correlated (band-like) functionals are more appropriate for low (high) pressures^47^. Thus, following this strategy, let us now turn our attention to the results obtained by B3PW91-$\alpha$= 0.10 and LDA XCFs, in addition to the results obtained by B3PW91-$\alpha$= 0.20. The 4f-Ce electrons have lowest degree of localization within the LDA XCF. Moreover, the degree of localization in the B3PW91 XCF can be controlled by the $\alpha$ parameter; lower $\alpha$ parameter is equivalent to lower degree of localization. Comparing the results of LDA XCF and B3PW91 with $\alpha$=20 and 0.10 reveal that reduction of the degree of localization for 4f-Ce electrons does not change the value of $S^{max-pos}$ drastically at very low temperatures and* P* = 0. However, this effect is temperature dependent and becomes more apparent as temperature rises. In fact at higher temperatures, reduction of the localization degree improves the $S^{max-pos}$ considerably. As an interesting point, the $S^{max-pos}$ within the lowest localized XCF, i.e., LDA, behaves the same as the Seebeck coefficient of normal metals; the $S^{max-pos}$ increases monotonically by increase of temperature and there is not any peak in it at all the considered temperature range. Based on this result, it seems that the considerable peak seen in thermopower of the most of the Ce-based compounds^22–24,26,28,49^ may be originated from the high degree of the 4f-Ce electrons. In contrast to the $S^{max-pos}$, the effect of the reduction of the localization degree on the $S^{max-neg}$ is not very noticeable at most of the considered temperatures, see Fig. 1bi1 for i = 1–3. Based on our results, the doping levels related to $S^{max-pos}$ are electrons at* P* = 0, independent of the used XCFs. The doping levels related to the $S^{max-neg}$ are holes at all the considered temperature range for B3PW91-$\alpha$ = 0.10 and LDA. This result holds for $\alpha$ = 0.20 up to about T = 50 K. But, at this temperature a gap occurs and the type of the doping levels related to $S^{max-neg}$ changes to electron. According to our results, applying pressure does not change the $S^{max-pos}$ drastically within the B3PW91-$\alpha$ = 0.10 and LDA, the same as the B3PW91-$\alpha$= 0.20 at very low temperatures. However, once temperature increases, the effect of pressure depends on the degree of localization (the used XCFs), as compared in Figs. 1ai1 for i = 1–3. This comparison shows that the most of the effect of pressure on the $S^{max-pos}$ is seen by the LDA functional. Our results also show that the effect of pressure on the $S^{max-neg}$ is strongly depend on the XCFs at most of the considered temperature range. This result holds for the doping levels related to both $S^{max-pos}$ and $S^{max-neg}$, as well. In summary, we conclude this section using these points that: (1) the $S^{max-pos}$ behavior of CeIn$_3$ depends on the used XCFs. It shows a peak at very low temperature using the high localized B3PW91-$\alpha$ = 0.20, which is a usual behavior for the thermopower in most of the Ce-based compounds. However, it behaves as a normal metal within the low localized LDA XCF. (2) The effects of pressure and degree of localization on the $S^{max-pos}$ depend upon the temperature; these effects are very small at very low temperatures, however, they become more important at higher temperatures. (3) The effect of localization degree on $S^{max-neg}$ is less than that on $S^{max-pos}$. (4) Imposing pressure affects $S^{max-neg}$ too, but these effects are not very noticeable. (5) The effects of pressure on the $S^{max-pos}$ and $S^{max-neg}$ strongly depend on the degree of localization. 6) The doping levels related to $S^{max-pos}$ and $S^{max-neg}$ are also affected by pressure and depend on the degree of localization.

**Figure 2:**
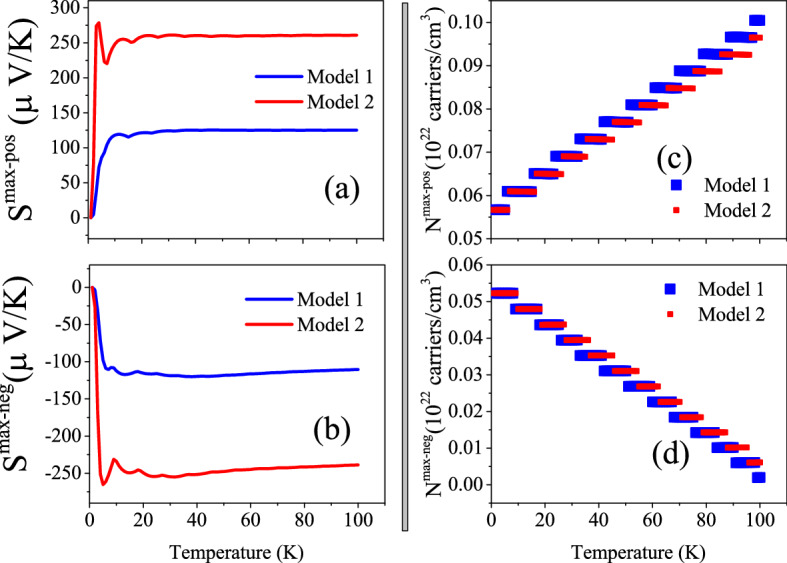
(**a**) Maximum positive or hole-like of spin up Seebeck coefficient ($S^{max-pos}$) and (**b**) maximum negative or electron-like of spin up Seebeck coefficient ($S^{max-neg}$) of hole/electron doped CeIn$_3$ versus temperature calculated by B3PW91-$\alpha =0.20$ at *P* = 0 using the $\tau =\tau _0 \epsilon ^{(r-1/2)}$ model with $\tau _0=5$, $r=2$ (Model1) and $r=4$ (Model2). Doping levels related to the (**c**) $S^{max-pos}$ and (**d**) $S^{max-neg}$.

The $S^{max-pos}$ and $S^{max-neg}$ are calculated based on the Model1 ($r=2$) and Model2 ($r=4$). The $S^{max-pos}$ and $S^{max-neg}$ together with their corresponding doping levels are presented in Fig. 2. The results, as shown in Fig. 2a, predict that $S^{max-pos}$ calculated by Model2 is more than two times greater than that calculated by Model1 for all the considered temperature range. This figure also reveals that the $S^{max-pos}$ increases as temperature increases up to about 10 K in both of the considered models. However, it is approximately independent of the temperature for T > 10 K. A short peak is predicted for $S^{max-pos}$ at T$\approx$ 10 K by Model2, while this is not the case for Model1. Similar trends are observed for the $S^{max-neg}$ values in Fig. 2b. Comparing Fig. 2a with Fig. 1a11 reveals that $S^{max-pos}$ predicted by the constant $\tau$ model is more than 10 (20) times smaller than that predicted by the Model1 (Model2) using B3PW91-$\alpha =0.20$ at *P* = 0. The same trends can be observed for $S^{max-neg}$ by comparing the Fig. 2b with Fig. 1b11.

### Electrical conductivity

**Figure 3:**
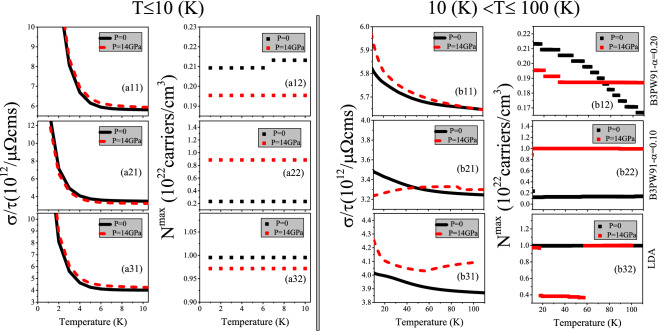
Maximum values of spin up electrical conductivity per relaxation time ($\tau$), i.e., $\sigma ^{max}/\tau$ of electron/hole doped CeIn$_3$ versus temperature for (**ai1**) T $\le$ 10 K and (**bi1**) 10 K < T $\le$ 100 K calculated using B3PW91 with $\alpha$  = 0.20, 0.10 and LDA for the *P* = 0 and 14 GPa pressures. Doping levels related to the $\sigma ^{max}/\tau$ are displayed in (**ai2**) and (**bi2**) panels for T $\le$ 10 K and 10 K < T $\le$ 100 K, respectively. The i-index varies from 1 to 3. In the AFM phase, spin down results are the same as spin up results.

Electrical conductivity, $\sigma (\mu ,~\text {T}$), measures the ability of a material for passing electricity. As expressed in Eq. 4, the electrical conductivity is a tensor with nine components. However, the same as Seebeck coefficient we need to investigate only one of the diagonal components of this tensor. The latter equation also shows that electrical conductivity is calculated as a function of relaxation time ($\tau$) within Botlzmann theory. Therefore, we investigate the electrical conductivity per relaxation time, i.e., $\frac{\sigma }{\tau }$. The maximum values of $\frac{\sigma }{\tau }$, i.e., $\frac{\sigma ^{max}}{\tau }$, calculated using the aforementioned XCFs are displayed in Fig. 3 versus temperature at *P* = 0 and 14 GPa pressures. The (ai1) [(bi1)] panels of this figure show the $\frac{\sigma ^{max}}{\tau }$ for T $\le$ 10 K [10 K < T $\le$ 100 K]. The doping levels corresponding to the $\frac{\sigma ^{max}}{\tau }$ are also displayed in the (ai2) and (bi2) panels of the latter figure for T $\le$ 10 K and 10 K < T $\le$ 100 K, respectively. The $\frac{\sigma }{\tau }$ values at specified temperatures T = 10, 50, and 100 K are also tabulated in Table 3. Based on our results independent of the used XCFs, the $\frac{\sigma ^{max}}{\tau }$ decreases with increase of temperature for T $\le$ 10 K at *P* = 0. The same result is also seen for 10 K < T $\le$ 100 K. However, comparing the (ai1) and (bi1) panels of Fig. 3 indicates that the rate of increase for temperatures lower than T $\simeq$ 5 K is considerably more than that for higher temperatures. This result is in accord with experimental results for electrical conductivity of several Ce-based compounds^40,79,80^. Our calculations show that the hole doping leads to the $\frac{\sigma ^{max}}{\tau }$ at all the considered temperature ranges at zero pressure within the three XCFs. Comparing the results for different pressures reveals that applying pressure does not change drastically the $\frac{\sigma ^{max}}{\tau }$ at all of the considered temperature. This result is confirmed by the data given in Table 3 for T = 10, 50, 100 K. Applying pressure also does not change the type of the doping levels related to $\frac{\sigma ^{max}}{\tau }$, but it changes their values considerably.

**Figure 4:**
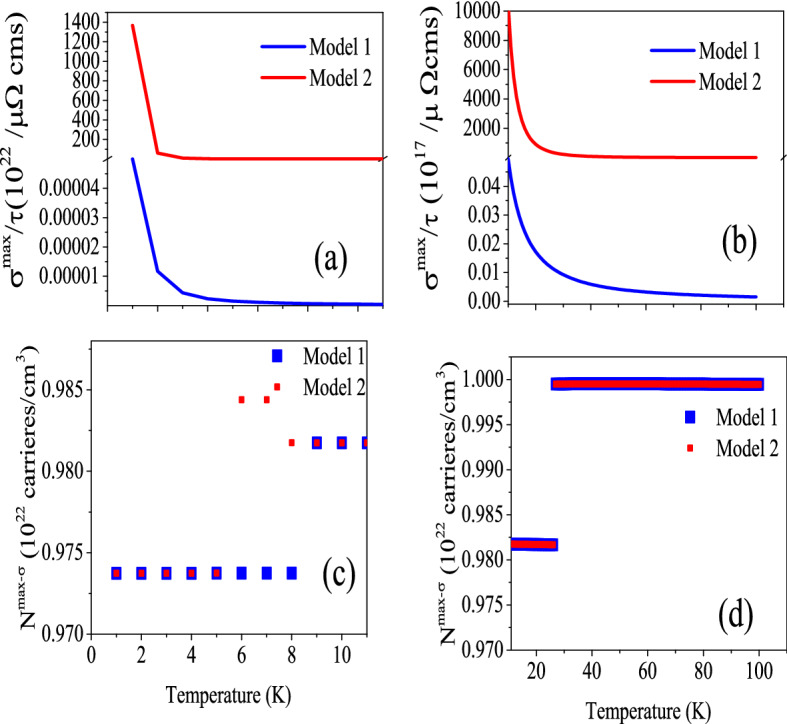
Maximum values of electrical conductivity of CeIn$_3$ per relaxation time, $\frac{\sigma ^{max}}{\tau }$, calculated using B3PW91-$\alpha =0.20$ at *P* = 0 employing the $\tau =\tau _0 \epsilon ^{(r-1/2)}$ model with $\tau _0=5$, $r=2$ (Model1) and $r=4$ (Model2) for (**a**)  0 < T $\leqslant$ 10 K and (**b**) 10 K < T $\leqslant$ 100 K temperature range. The related doping levels are displayed in the (**c**) and (**d**) panels, respectively.

Our results reveal that the effects of pressure on the values of doping levels can strongly depend on the used XCF. Similar results can be seen for the $\frac{\sigma ^{max}}{\tau }$, i.e., the effects of pressure on the $\frac{\sigma ^{max}}{\tau }$ are also related to the used XCFs. Different functionals have been used to account for different degrees of 4f localization. One would naturally expect that the electric, transport, and the other quantities vary from LDA over $\alpha =0.10$ to 0.20 continuously or show at least an expected trend. However, e.g., for the conductivity, as shown in Fig. 3, the pressure dependence for LDA and B3PW91 with $\alpha =0.20$ is quite similar, whereas the intermediate value unexpectedly sticks out. A careful analysis elucidates that the former similar pressure dependence and the latter unexpected behavior originate from the different effects of pressure on the band structure of CeIn$_3$ using B3PW91 with $\alpha =0.10$ compared to B3PW91 with $\alpha =0.20$ and LDA. To this end, let us below consider our results reported in Table 3 together with Figs. 4 and 5 of Ref. ^47^. For the three bands that crossed the Fermi level at zero pressure, as indicated by $\gamma _1$, $\gamma _2$, and $\gamma _3$ bands in Fig. 4 of Ref.^47^, the occupancy numbers were calculated by the LDA, B3PW91 with $\alpha = 0.10$ and $\alpha = 0.20$ XCFs at both zero and 14 GPa pressures, see the last two columns of Table 3 of Ref.^47^. In the latter Table, the occupation numbers of the fully (partially) occupied bands were reported to be 1.000 (less than unity). The number of bands that crossed the Fermi level remained unchanged after imposing pressure using B3PW9 with $\alpha =0.20$ and LDA, while it was reduced from 3 bands at zero pressure to 2 bands at *P* = 14 GPa using B3PW91-$\alpha =0.10$, as would be interestingly observed from the occupancy numbers presented in Table 3 of Ref.^47^. Therefore, the trend of the physical quantities could be accordingly changed (unaffected) using the intermediate value of $\alpha = 0.10$ (larger value of $\alpha = 0.20$ and LDA) by the pressure applied. These different behaviors would be also consistently seen in the Fermi surfaces of CeIn$_3$, as shown in Fig. 5 of Ref.^47^. This verifies that the unexpected trend of the intermediate value of $\alpha = 0.10$ can be attributed to the reduction of the number of partially occupied bands after applying pressure by taking band structures, and Fermi surfaces, as well as the occupation numbers of the three $\gamma _1$, $\gamma _2$, and $\gamma _3$ bands into account simultaneously.

Our results also reveal that $\frac{\sigma ^{max}}{\tau }$ is affected by the degree of localization, but this effect is very small; the $\frac{\sigma ^{max}}{\tau }$ using B3PW91-$\alpha$ = 0.20 is slightly higher than those obtained from B3PW91-$\alpha$ = 0.10 and LDA. The Table 3 reveals this result for T = 10, 50, 100 K. However, the effect of localization degree considerably affects the doping levels, see (ai2) panels of Fig. 3 for T $\le$ 10 K. The same result can be seen for 10 K < T $\le$ 100 K by comparing the Figure 3bi2.

**Table 3:** The maximum values of electrical conductivity per relaxation time, $\sigma ^{max}/\tau$, and electronic part of thermal conductivity per relaxation time, $\kappa _e^{max}/\tau$, of CeIn$_3$ at *P* = 0 and 14 GPa using the B3PW91 XCF with $\alpha$ = 0.20, 0.10 and LDA XCF at specified temperatures T = 10, 50, and 100 K.

T(K)	B3PW91-$\alpha$ = 0.20		B3PW91-$\alpha$ = 0.10		LDA	
	*P* = 0	*P* = 14 GPa	*P* = 0	*P* = 14 GPa	*P* = 0	*P* = 14 GPa
$$\boldsymbol{\sigma} ^{\user2{max}}/\boldsymbol{\tau}$$						
10	5.82	5.95	3.49	3.23	4.02	4.26
50	5.69	5.71	3.31	3.32	3.92	4.04
100	5.65	5.65	3.25	3.30	3.87	4.09
$$\boldsymbol{\kappa}_{\textbf{e}}^{\user2{max}}/ \boldsymbol{\tau}$$						
10	1.41	1.43	0.84	0.80	0.97	1.00
50	6.92	6.90	4.03	4.09	4.67	5.10
100	13.60	13.70	7.84	8.09	9.33	10.21

The units of $\sigma ^{max}/\tau$ and $\kappa _e^{max}/\tau$ are $\frac{10^{14}}{\mu \Omega m s}$ and $\frac{10^{12}W}{cm K s}$, respectively.

Besides the constant $\tau$ model, we calculate the $\frac{\sigma ^{max}}{\tau }$ using Model1 and Model2. The calculated $\frac{\sigma ^{max}}{\tau }$ along with its related doping levels are presented in Fig. 4. The Fig. 4a [Fig. 4b] shows the $\frac{\sigma ^{max}}{\tau }$ for T$\le$ 10 K [T>10 K]. Similar to the Seebeck coefficient, the values of $\frac{\sigma ^{max}}{\tau }$ predicted by Model2 are considerably larger than those predicted by the Model1 at all the considered temperature ranges. Comparing the Figs. 4a and b with Fig. 3a11 and b11 reveals that the $\frac{\sigma ^{max}}{\tau }$ behavior predicted by Model1 and Model2 is the same as the that predicted by the constant $\tau$ model at *P* = 0 using B3PW91-$\alpha =0.20$. However, the values of $\frac{\sigma ^{max}}{\tau }$ predicted by Model1 and Model2 are considerably larger than those predicted by the constant $\tau$ model.

### Thermal conductivity

**Figure 5:**
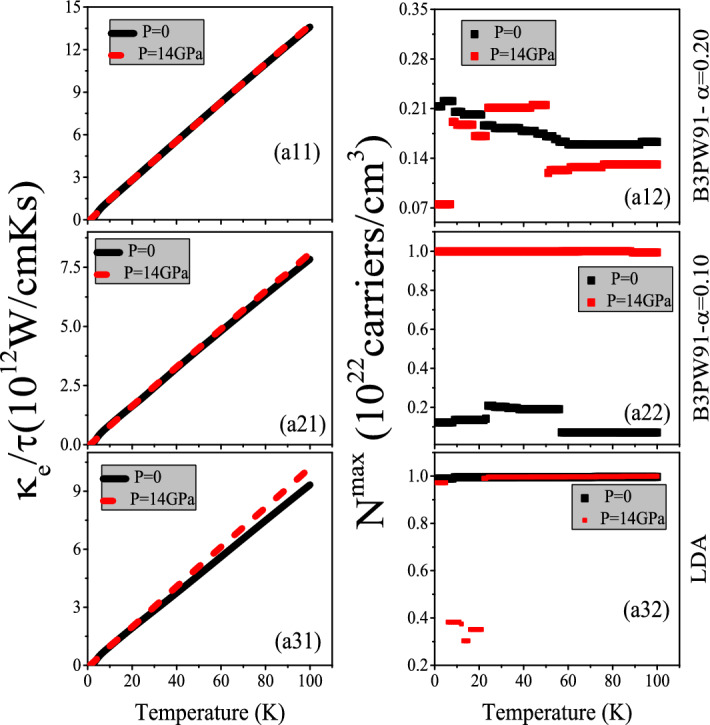
(**ai1**) Maximum values of spin up electronic thermal conductivity per relaxation time ($\tau$), i.e., $\kappa ^{max}_{e}/\tau$, of electron/hole doped CeIn$_3$ versus temperature for T $\le$ 100 K calculated using B3PW91 with $\alpha$  = 0.20, 0.10 and LDA for *P* = 0 and 14 GPa pressures. Doping levels related to $\kappa _e^{max}/\tau$ are displayed in the (**ai2**) panels. In the AFM phase, spin down results are the same as spin up results.

**Figure 6:**
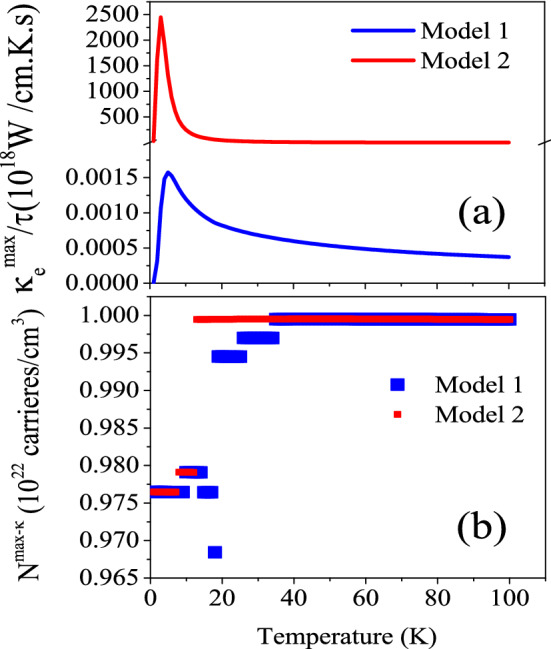
Maximum values of electrical portion of thermal conductivity of CeIn$_3$ per relaxation time, $\frac{\kappa _e^{max}}{\tau }$, calculated by B3PW91-$\alpha =0.20$ at *P* = 0 using the $\tau =\tau _0 \epsilon ^{(r-1/2)}$ model with $\tau _0=5$, $r=2$ (Model1) and $r=4$ (Model2) for T $\leqslant$ 100 K temperature range. The related doping levels are displayed in the (**b**) panel.

Thermal conductivity ($\kappa$) is another factor which can affect the efficiency of a thermoelectric material. This property of a thermoelectric material is related to its ability of transferring heat^81^. In principle, both phonons and electrons can contribute to the thermal conductivity. The phonons part of thermal conductivity in semiconductors is prominent while the electronic part is very small. In metals, unlike semiconductors, the lattice part constitutes a very small fraction of the total thermal conductivity and therefore can be safely neglected. It is smaller than 2 percent^82,83^ for metals. Therefore, we focus on the electronic part of the thermal conductivity ($\kappa _\text {e}$) for the present intermetallic compound. The electronic part of the thermal conductivity is a tensor of rank 2 with nine components, see Eq. 5. However, the same as the electrical conductivity and Seebeck coefficient tensors, it is enough to consider only one of the diagonal components of this tensor, here.

Furthermore, the same as the electrical conductivity, the $\kappa _e$ is a function of relaxation time ($\tau$) in the Boltzmann theory, see Eq. 5. Thus, we discuss the electronic part of thermal conductivity per relaxation time. The maximum values of the electronic part of thermal conductivity per relaxation time, i.e., $\frac{\kappa _e^{max}}{\tau }$, calculated using the aforementioned XCFs are shown in the (ai1) panels of Fig. 5 (for i = 1–3) as function of temperature at zero and 14 GPa pressures. The $\frac{\kappa _e^{max}}{\tau }$ values at the specified temperatures T = 10, 50, and 100 K are also tabulated in Table 3. The (ai2) panels of the latter figure show the doping levels related to the $\frac{\kappa _e^{max}}{\tau }$. As shown in Fig. 5ai1, the $\frac{\kappa _e^{max}}{\tau }$ is increased by increase of temperature. This result is independent of the used XCFs and applied pressures. Our results also show that the effects of pressure on $\frac{\kappa _e^{max}}{\tau }$ are negligible at all the considered temperature ranges within the utilized XCFs. Similar result can be observed for the degree of 4f-Ce localization by comparing Fig. 5ai1 for i = 1–3; the effects of the localization degree on $\frac{\kappa _e^{max}}{\tau }$ are negligible at all the considered temperature ranges independent of the applied pressures. These results can be seen from the Table 3 for T = 10, 50, 100 K. On the contrary, the doping levels related to the $\frac{\kappa _e^{max}}{\tau }$ are affected considerably by pressure or localization degree, as can be seen clearly from the (ai2) panels of Fig. 5. As an interesting point, the effect of pressure on doping levels strongly depends on the used XCFs, as predicted in our previous work^47^.

Besides the constant $\tau$ model, the $\frac{\kappa _e^{max}}{\tau }$ is calculated by Model1 and Model2 at *P* = 0 using B3PW91-$\alpha =0.20$, as presented in Fig. 6a. The doping levels related to $\frac{\kappa _e^{max}}{\tau }$ is shown in Fig. 6b. The comparison of the Figs. 6a and 5a11 reveals that the behavior of $\frac{\kappa _e^{max}}{\tau }$ predicted by Model1 and Model2 is very different from that predicted by the constant $\tau$ model. As shown in Fig. 6a, the Model1 and Model2 predict a peak for $\frac{\kappa _e^{max}}{\tau }$ at T $\approx$ 3 K. After the latter temperature, the $\frac{\kappa _e^{max}}{\tau }$ decreases as temperature increases. Moreover, the $\frac{\kappa _e^{max}}{\tau }$ predicted by Model1 and Model2 is considerably larger than that predicted by the constant $\tau$ model. In addition, the same as Seebeck coefficient and electrical conductivity, the $\frac{\kappa _e^{max}}{\tau }$ predicted by Model2 is considerably more than that predicted by Model1 at all the considered temperature ranges.

### Power factor

**Figure 7:**
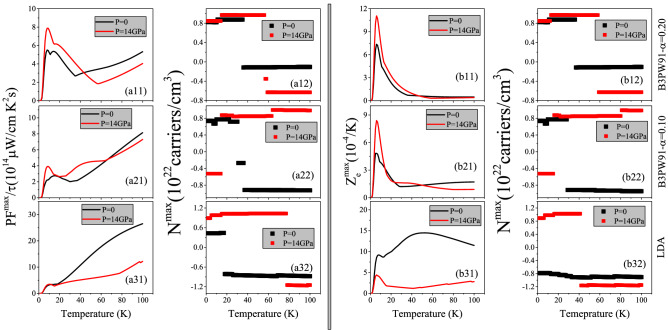
(**ai1**) Maximum values of spin up power factor per relaxation time, PF$^{max}/\tau$, versus temperature calculated using B3PW91 with $\alpha$  = 0.20, 0.10 and LDA for the *P* = 0 and 14 GPa pressures. (**ai2**) Doping levels related to the PF$^{max}/\tau$ . (**bi1**) Maximum values of spin up electronic figure of merit, $Z_e^{max} = \frac{\sigma S^2}{\kappa _e}$, versus temperature calculated using B3PW91 with $\alpha$  = 0.20,0.10 and LDA for the *P* = 0 and 14 GPa pressures. (**bi2**) Doping levels related to the $Z_e^{max}$. The i-index varies from 1 to 3. In the AFM phase, spin down results are the same as spin up results.

The Eq. 3 implies that the Seebeck coefficient is inversely proportional to the electrical conductivity so that when the Seebeck coefficient increases, the electrical conductivity decreases and vice versa. Furthermore, based on the *ZT* formula, i.e., $ZT=\frac{\sigma S^2}{\kappa }$, to enhance the thermoelectric efficiency, both of the Seebeck coefficient and electrical conductivity play important roles and thence both of them should be considered simultaneously. In other words, the numerator of *ZT*, i.e., $\sigma S^2$, which is called power factor (PF) would be investigated instead of *S* or $\sigma$. This is the goal of this section. In this section, we aim to investigate the response of PF to the hole/electron doping and hydrostatic pressure as well as the change of the localization degree for CeIn$_3$ compound. The PF is a tensor of rank two with nine components. However, similar to $S(\mu , \text {T})$, $\sigma (\mu , \text {T})$ and $\kappa (\mu , \text {T})$ tensors, it is sufficient to investigate only one of its diagonal elements for CeIn$_3$. As expressed in Eq. 4, the $\sigma (\mu , \text {T})$ is a function of the relaxation time ($\tau$). Therefore, the PF is a function of $\tau$, too. The maximum values of the diagonal PF element per $\tau$, i.e., PF$^{max}/\tau$ versus temperature are shown in the (ai1) panels of Fig. 7 for the considered XCFs and pressures. The (ai2) panels of the latter figure display the doping levels related to the PF$^{max}/\tau$. As shown in Fig. 7a11, the high localized B3PW91-$\alpha$ = 0.20 XCF predicts that PF$^{max}/\tau$ occurs at very low temperatures (T $\simeq$ 10 K) and *P* = 0. This peak is also seen in the B3PW91-$\alpha$ = 0.10 XCF and *P* = 0. However, the value of this peak using $\alpha$ = 0.10 is considerably lower than $\alpha$ = 0.20. If we use the low localized LDA XCF, the peak of PF$^{max}/\tau$ at low temperatures completely disappears. Our results show that PF$^{max}/\tau$ increases monotonically as temperature increases using LDA XCF, as shown in Fig. 7a31. Comparing the results of different XCFs reveals that the effect of the localization degree on the PF$^{max}/\tau$ is temperature dependent. At very low temperatures, i.e., around T = 10 K, the reduction of the localization degree makes PF$^{max}/\tau$ worse. However, this effect is reversed and the PF$^{max}/\tau$ is improved by the reduction of the localization degree as temperature increases. Comparing the Fig. 7ai2 for i = 1–3 reveals that at fixed pressure, the doping levels related to PF$^{max}/\tau$ depend on the used XCFs, too. As shown in Fig. 7ai2, for very low temperatures the hole doping leads to the PF$^{max}/\tau$ at zero pressure using the considered XCFs, but the value of doping levels depends on the used XCFs. The Fig. 7ai2 also show a gap in the doping levels as temperature increases and thereby the type of the doping levels is changed to electron. This result is independent of the used XCFs. However, the values of the electron doping levels and the gap as well as the temperature (where this gap occurs) depend on the used XCF.

The PF$^{max}/\tau$ can be also affected by pressure. Based on our calculations using B3PW91 XCF with $\alpha$  = 0.20 ($\alpha$  = 0.10), applying pressure improves PF$^{max}/\tau$ for temperature lower than T$\simeq$ 45 K (T$\simeq$ 70 K ), while makes it worse for higher temperatures. The same result is obtained using LDA, but at T$\simeq$ 12 K; applying pressure improves PF$^{max}/\tau$ for temperature lower than T$\simeq$ 12 K, while makes it worse for higher temperatures using LDA. Interestingly, the value of improvement or deterioration of PF$^{max}/\tau$ due to applying pressure is strongly XCF dependent. We also see that the effect of pressure on the doping levels is strongly XCF dependent, see Fig. 7ai2 for i = 1–3. These results are in complete accord with our previous results^47^.

**Figure 8:**
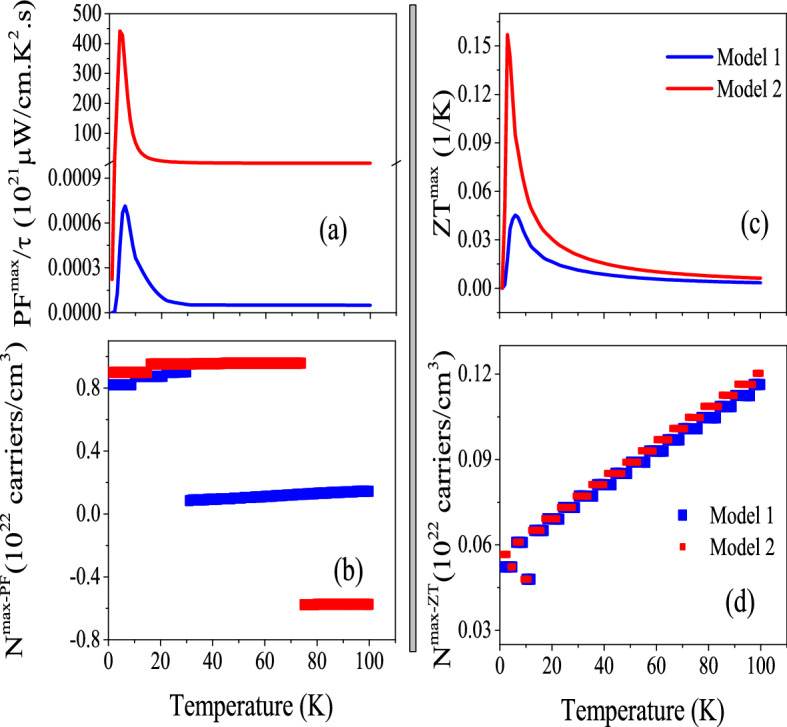
Maximum values of (**a**) power factor per relaxation time $\frac{PF^{max}}{\tau }$ and (**c**) electrical Z ($Z_e^{max}$) of CeIn$_3$ calculated by B3PW91-$\alpha =0.20$ at *P* = 0 using the $\tau =\tau _0 \epsilon ^{(r-1/2)}$ model with $\tau _0=5$, $r=2$ (Model1) and $r=4$ (Model2) for T $\leqslant$ 100 K temperature range. The corresponding doping levels are shown in (**b**) and (**d**).

In addition to the constant $\tau$ model, we calculated the PF$^{max}/\tau$ using the Model1 and Model2. The results is presented in Fig. 8a. The same as conductivity parameters, the PF$^{max}/\tau$ calculated by Model2 is considerably greater than that calculated by Model1 in order of magnitude at all the considered temperature ranges, as shown in Fig. 8a. This figure also shows that there is a peak in PF$^{max}/\tau$ calculated by the two considered models at very low temperatures. The comparison of Figs. 8a and 7a11 reveals that the behavior of PF$^{max}/\tau$ calculated by Model1 and Model2 is very similar to that calculated by the constant $\tau$ model, however, its orders of magnitude as predicted by Model1 and Model2 are significantly more than that predicted by the constant $\tau$ model.

**Figure 9:**
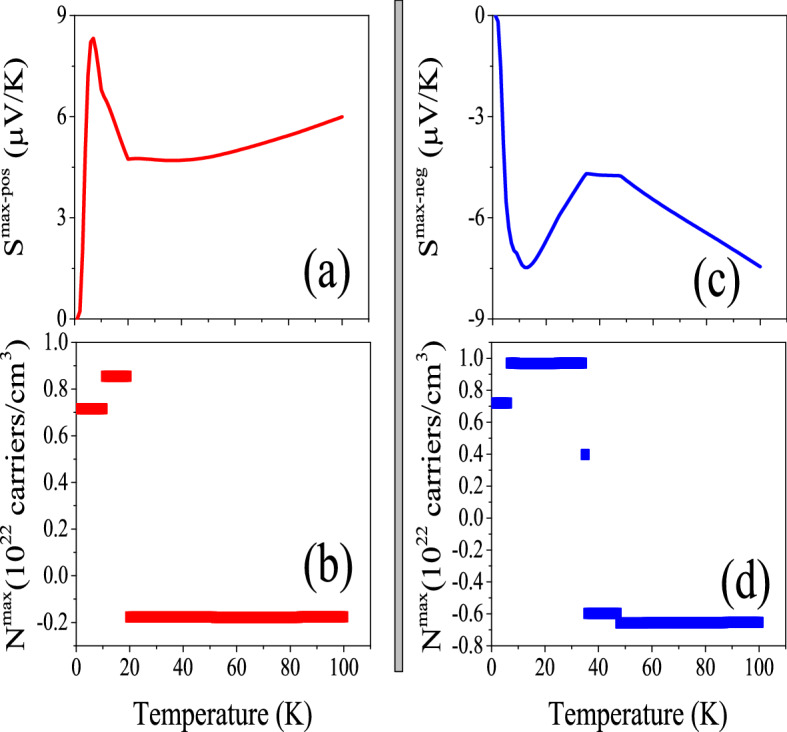
Maximum positive or hole-like of spin up Seebeck coefficient ($S^{max-pos}$) and (**b**) maximum negative or electron-like of spin up Seebeck coefficient ($S^{max-neg}$) of hole/electron doped CeIn$_3$ versus temperature calculated by GGA+U with $\text {U}_{\text {eff}}=5.5~\text {eV}$ for the *P* = 0 pressure. Doping levels related to the $S^{max-pos}$ and $S^{max-neg}$ are displayed in the (**c**) and (**d**) panels, respectively.

**Figure 10:**
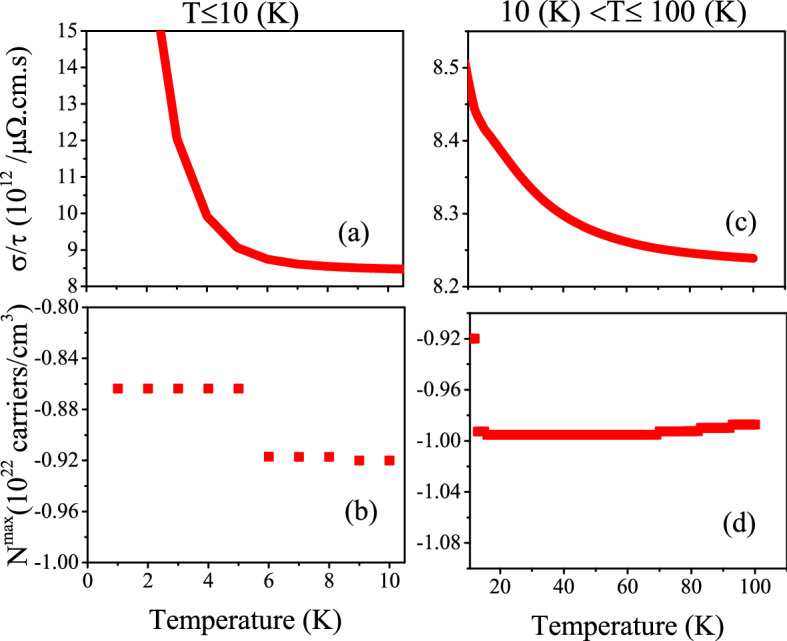
Maximum values of spin up electrical conductivity per relaxation time ($\tau$), i.e., $\sigma ^{max}/\tau$ of electron/hole doped CeIn$_3$ versus temperature for (**a**) T $\le$ 10 K and (**b**) 10 K < T $\le$ 100 K calculated using GGA+U with $\text {U}_{\text {eff}}=5.5~\text {eV}$ for the *P* = 0 pressure. Doping levels related to the $\sigma ^{max}/\tau$ are displayed in the (**c**) and (**d**) panels for T $\le$ 10 K and 10 K < T $\le$ 100 K, respectively.

In fact, the efficiency of thermoelectricity depends on both PF and thermal conductivity. As stated above, we only calculate the electronic part of thermal conductivity, i.e., $\kappa _e$, here. This means that the calculated figure of merit, *Z*, is not exact. Therefore, we introduce the electronic figure of merit as $Z_e=\frac{\sigma S^2}{\kappa _e}$ to emphasize that the lattice portion of thermal conductivity is neglected. It is obvious that $Z_e$ and *Z* are not exactly the same as each other. However, in metals the lattice part is very small and can be safely neglected^82,83^, specifically at low temperatures. Thus, $Z_e$ is approximately very close to *Z*. The (bi1) panels of Fig. 7 display the maximum values of $Z_e$, i.e., $Z_e^{max}$ versus temperature using the aforementioned XCFs and pressures. The (bi2) panels show the doping levels related to $Z_e^{max}$. As the Fig. 7b11 reveals, the B3PW91 XCF with $\alpha$ = 0.20 predicts a remarkable peak for $Z_e^{max}$ at very low temperatures and *P* = 0. The same result is predicted using B3PW91 with $\alpha$ = 0.10, see the Fig. 7b21. We also see a local maximum value for $Z_e^{max}$ at very low temperatures using the LDA XCF at *P* = 0, however, the global maximum occurs around the T = 50 K. Our results show that imposing pressure increases the value of the discussed peaks considerably using B3PW91 XCF with $\alpha$  = 0.20, and 0.10. However, this is not the case, if we use LDA XCF. As the Fig. 7b31 shows, imposing pressure considerably decreases the value of $Z_e^{max}$ using LDA. These results confirm that the effect of pressure depend on the considered degree of localization. Our results reveal that the peaks of $Z_e^{max}$ within B3PW91-$\alpha$ = 0.20 XCF at both considered pressures are originated from the hole doping, see Fig. 7b12. The same result is seen for $\alpha$ = 0.10 at zero pressure. But for *P* = 14 GPa, the electron doping leads to the $Z_e^{max}$. The electron doping calculated by LDA XCF is related to the $Z_e^{max}$ at all the considered temperature ranges at zero pressure, as shown in Fig. 7b31. However, the peak of the $Z_e^{max}$ at *P* = 14 GPa originates from the hole doping using the LDA, in contrast to the zero pressure. Based on these results it can be concluded that the effect of pressure on $Z_e^{max}$ and its doping levels strongly depends on the localization degree considered for the 4f-Ce electrons. The calculated $Z_e^{max}$ using Model1 and Model2 is represented in Fig. 8c. Comparing the latter figure with Fig. 7b11 reveals that the Model1 and Model2 predict higher $Z_e^{max}$ for CeIn$_3$ than the constant $\tau$ model. Moreover, the peak of the $Z_e^{max}$ in Model2 is about 4 times of Model1.

## Paramagnetic phase

The CeIn$_3$ compound is an antiferromagnetic (AFM) system at ambient pressure with a Néel temperature of T$_N\approx$10.1 K^43^. Thus, this compound lies in its AFM phase below the Néel temperature, while it transits to its paramagnetic (PM) phase above 10.1 K at ambient pressure. We have already studied thermoelectric properties of this system at its AFM phase from zero to 100 K in previous sections. In the previous thermoelectric sections, however, we have not considered the phase transition from AFM phase to PM phase. Thus, a question can be naturally raised on the validity of our results for the temperatures higher than the Néel temperature of this system. This question is raised due to the fact that the behavior of the 4f-spins can be different at lower temperatures as compared to higher temperatures than T$_N$. The question can be how accurate the effect of temperature is captured in our study. In order to estimate the validity of our results, we have considered and set up the PM phase, as discussed in “Calculation details” section. The considered angles of the 4 Ce atoms in the $2 \times 2 \times 1$ supercell are tabulated in Table 4. With these considered angles, the total magnetic moment of the supercell is zero, as should be in PM phase.

**Figure 11:**
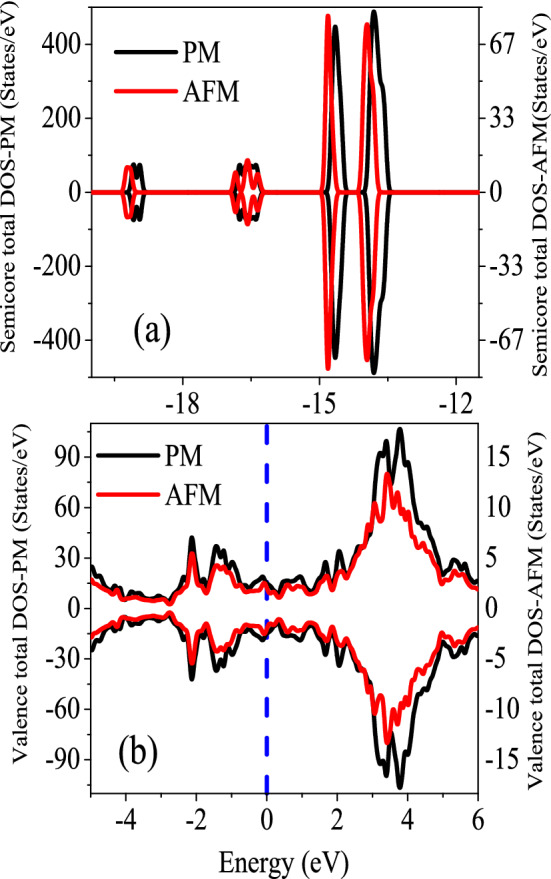
Total DOSs of CeIn$_3$ in (**a**) semicore and (**b**) valence regions at *P* = 0 calculated using GGA+U with $\text {U}_{\text {eff}}=5.5~\text {eV}$ at paramagnetic (PM) and antiferromagnetic (AFM) phases. The vertical dash line in figure (**b**) indicates the Fermi level.

**Table 4:** Polar angles ($\phi , \theta$) which determine the spin direction of the 4 Ce atoms in $2\times 2\times 1$ supercell of CeIn$_3$ used for noncollinear magnetism calculations using WIENncm code.

Atom	$\phi$	$\theta$
Ce1	0	30
Ce2	90	30
Ce3	180	150
Ce4	270	150

Due to the practical restriction discussed in “Calculation details” section, we have performed the calculations in the PM phase using GGA+U approach with $\text {U}_{\text {eff}}=5.5~\text {eV}$ instead of the more successful hybrid B3PW91 functional with $\alpha = 0.2$. The total and partial DOSs of CeIn$_3$ in the PM phase are shown in Fig. 11. For comparison we have also included DOSs of the AFM phase in this figure. Although for the AFM phase there is not practical limitation to include the B3PW91 functional with $\alpha = 0.2$, the AFM calculations are also performed using GGA+U approach with$\text {U}_{\text {eff}}=5.5~\text {eV}$ to be more comparable with the PM results. This figure clearly shows that the difference between AFM and PM DOSs is not too much; the PM and AFM DOSs are close to each other.

**Figure 12:**
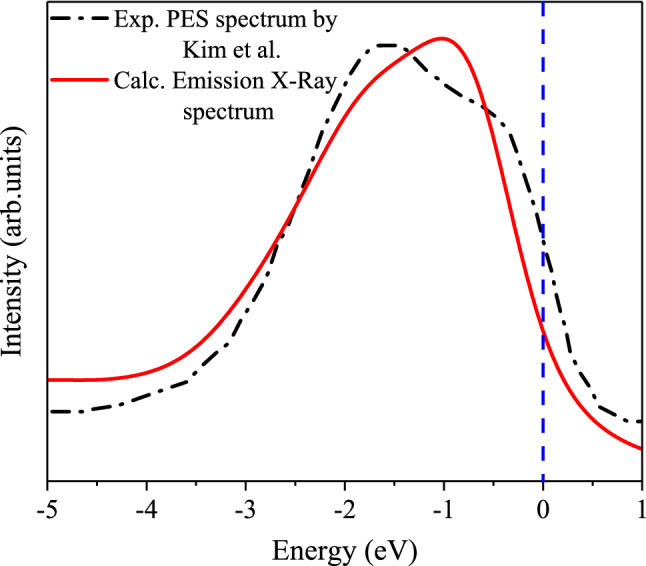
Theoretical emission X-Ray spectrum calculated by GGA+U with $\text {U}_{\text {eff}}=5.5~\text {eV}$ for theAFM phase of CeIn$_3$ along with the experimental photoemission spectroscopy (PES) spectrum^84^.The vertical dash line in figure indicates the Fermi level.

Moreover, we have calculated the X-Ray spectrum for the AFM phase of our compound, as shown by a solid red curve in Fig. 12. In order to estimate the accuracy of our calculations, the experimental photoemission spectroscopy (PES) spectrum, as measured by Kim and coworkers^84^, are also included, as shown by dash-dotted blue curve in Fig. 12. The comparison of the theoretical an experimental spectra shows that there is an agreement between our prediction made by GGA+U approach with $\text {U}_{\text {eff}}=5.5~\text {eV}$ and the experimental PES spectra.

**Figure 13:**
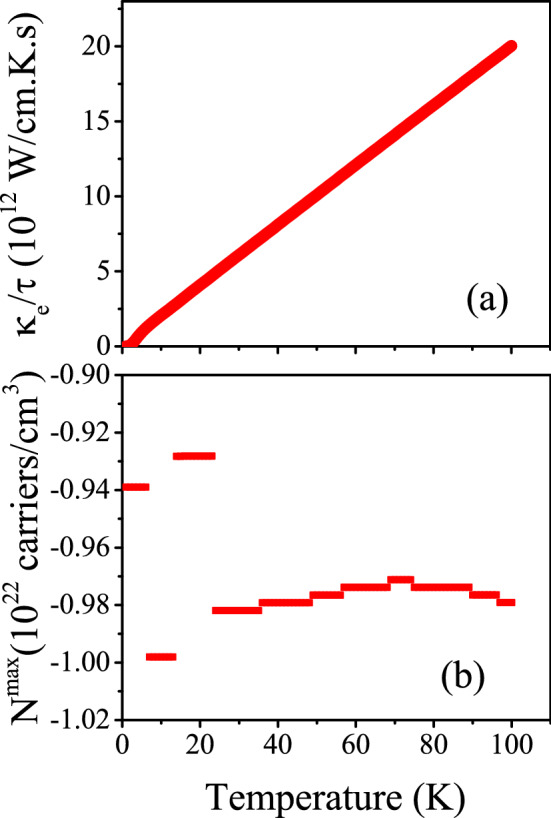
(**a**) Maximum values of spin up electronic thermal conductivity per relaxation time ($\tau$), i.e., $\kappa ^{max}_{e}/\tau$, of electron/hole doped CeIn$_3$ versus temperature for T $\le$ 100 K calculated using GGA+U with $\text {U}_{\text {eff}}=5.5~\text {eV}$ for the *P* = 0 pressure. Doping levels related to the $\kappa _e^{max}/\tau$ are displayed in the (**b**) panel.

**Figure 14:**
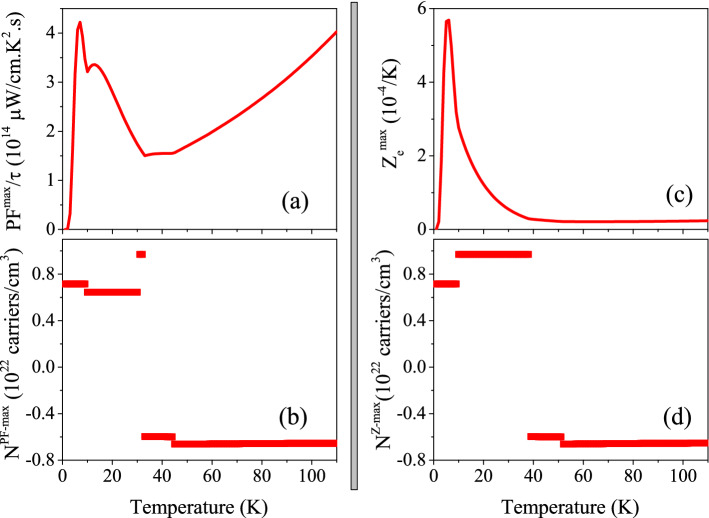
Maximum values of (**a**) power factor per relaxation time (PF$^{max}/\tau$) and (**b**) electronic figure of merit (Z$_e^{max}$) versus temperature for $T \le 100~\text {K}$ calculated by GGA+U with $\text {U}_{\text {eff}}=5.5~\text {eV}$ at *P* = 0 pressure. Doping levels related to the PF$^{max}/\tau$ and $Z_e^{max}$ are displayed in (**c**) and (**d**), respectively.

Unfortunately, the current version of the BoltzTraP code cannot use the outputs of the WIENncm code. However, based on the similarity of the DOSs in the AFM and PM phases, as shown in Fig. 11, one may expect that the thermoelectric results of the AFM phase may not be very far from those of the PM phase. Therefore, in anticipation of further investigations, we have limited the accuracy of our results to the outputs of the AFM phase and calculate the thermoelectric properties of CeIn$_3$. The calculated thermoelectric properties are also shown for the AFM phase using GGA+U with $\text {U}_{\text {eff}}=5.5~\text {eV}$ in the Figs. 9, 10, 11, 12, 13 and 14. Since the discussions of the latter thermoelectric figures using GGA+U for the AFM and PM phases are similar to our previous discussions presented in the last thermoelectric sections using LDA and hybrid B3PW91 functionals for the AFM phase, we avoid to repeat them here.

## Phonon dispersion curves and DOS

**Figure 15:**
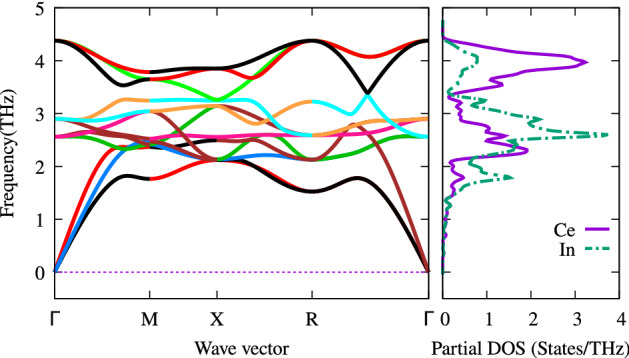
Phonon bandstructure and partial density of states of CeIn$_3$ at zero pressure. The solid (dashed) line denote the Ce (In) PDOS.

**Figure 16:**
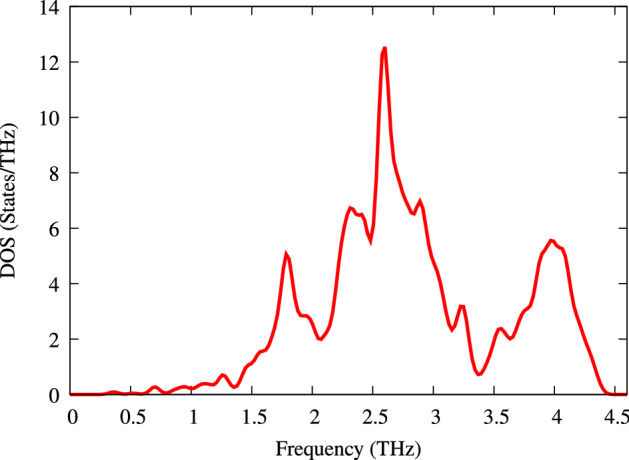
Phonon total DOS of CeIn$_3$ at zero pressure.

We have calculated the phonon band structure along with the corresponding phonon partial- and total-DOSs. The technical setup is discussed in “Calculation details” section. To this end, the scattering process is considered as Umklapp- or U-phonon-phonon scattering, phonon-electron scattering, and phonon-impurity scattering. Each scattering is characterized by a relaxation rate ($\frac{1}{\tau _c}$) which is the inverse of the corresponding relaxation time as follows:

8 $$\begin{aligned} \frac{1}{\tau _c}=\frac{1}{\tau _U}+\frac{1}{\tau _{ph-el}}+\frac{1}{\tau _M}, \end{aligned}$$

where $\frac{1}{\tau _U}$ is related to Umklapp, $\frac{1}{\tau _{ph-el}}$ is related to the phonon-electron and $\frac{1}{\tau _M}$ is related to phonon-impurity scatterings. As would be seen, the relaxation time now depends on the temperature and it is no longer fixed. By this, we have included Umklapp-phonon-phonon scattering, phonon-electron scattering, and phonon-impurity scattering. In this way, the relaxation time is made temperature dependent which is no longer fixed. In this paper, to anticipate further investigations on the thermoelectric properties of the system including all the above scattering processes, however, we only concentrate on the phonon band structures and phonon partial DOSs, as calculated in the presence of the above scattering processes and shown in Fig. 15. The total phonon DOS is also shown in the Fig. 16. As shown in these figures, the phonon bands and DOSs are mostly seen between $\omega =1.5-4.5$ THz. Because all the phonon frequencies are real, the crystal is dynamically stable at its equilibrium state. The dispersions of the transverse and longitudinal acoustic and optical modes can be clearly observed in the dispersion curves. The only symmetry band structure path in this compound is $\Gamma$-M-X-R-$\Gamma$, which means, on average, that the dispersion in $x-$,$y-$ and $z-$directions are the same. Therefore, it is expected that similar interactions occur in these symmetrical directions. This implies that the thermal conductivity is similar in the $x-$, $y-$ and $z-$directions. As can be seen in the phonon band structure, at about the frequency of 2.5 THz, there is one flat band near the M-X-R band path. This flat band, which corresponds to the peaks in the phonon PDOS, indicates that their corresponding states are localized, i.e., they behave like *“atomic states”*. As shown in Fig. 15, the peaks at moderate frequencies are composed of states of In atoms and a smaller fraction of states of Ce atoms. At high frequency, the peaks originate from the Ce states with some hybridization by In atoms. Moreover, at low frequencies between the R-$\Gamma$ band path two bands of the acoustic modes coincide with each other. The coincidence means that the transverse acoustic modes are the same and the displacement of both atoms has the same amplitude, direction and phase in this path.

## Kondo effect

**Figure 17:**
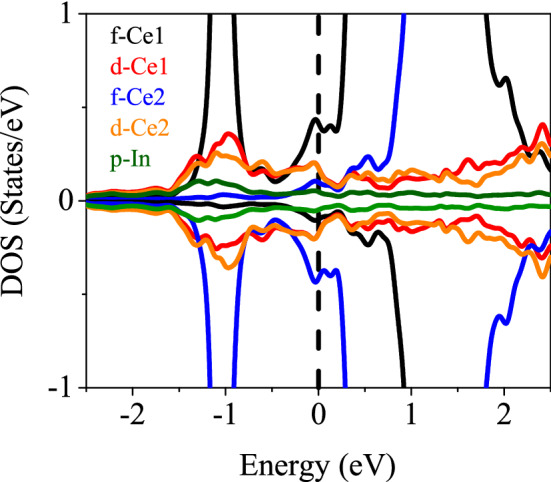
Partial DOSs of CeIn$_3$ calculated by B3PW91-$\alpha =0.20$ at *P* = 0. The vertical dashed line indicates the Fermi level.

**Figure 18:**
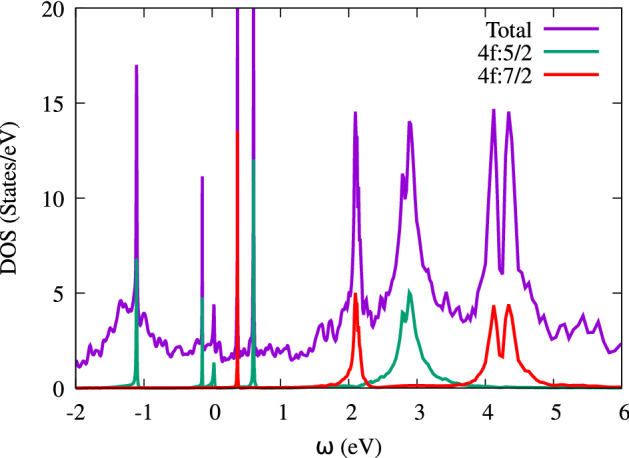
Total and partial DOSs of CeIn$_3$ calculated by LDA+DMFT at *P* = 0.

To close this paper, it is worth mentioning that there is a competition between Ruderman–Kittel–Kasuya–Yoshida (RKKY) interaction and Kondo effect in the Ce-based compounds. To determine the winner of this competition, their energies should be considered. Both of these energies depend on the exchange integral, $J_{cf}$^85^:

9 $$\begin{aligned} J_{cf}\propto \frac{V_{cf}^2}{E_F-E_f}, \end{aligned}$$

where $E_f$ is energy of 4f-Ce states, $E_F$ is the Fermi energy and $V_{cf}$ is the hybridization energy between 4f-Ce states and conduction electrons. To this end, we have calculated the partial DOSs of CeIn$_3$ for 4f and conduction states near the Fermi level using the B3PW91-$\alpha =0.20$ at *P* = 0 in the AFM phase. The result is shown in Fig. 17. This figure shows the strong hybridization between 4f-Ce state with conduction states, i.e., d-Ce and p-In. Based on this result the Kondo effect is expected for the case under study. Moreover, we have performed the DFT+DMTF calculations for CeIn$_3$ at *P* = 0, as discussed in“Calculation details” section. The total and partial DOSs of CeIn$_3$ are calculated by the DFT+DMTF using the imaginary part of retarded Greens functions, as shown in Fig. 18. The DFT+DMTF results show that there is a considerable peak in the total DOS near the $\omega =0$. This is an indication for the Kondo resonance in the case under study. Thus, our DFT+DMTF results also confirm the prediction of our hybrid results calculated by B3PW91-$\alpha =0.20$.

## Summary, outlook and disclaimer

In this paper, we have studied the influences of the pressure and 4f-localization degree on the thermoelectric efficiency of the electron/hole doped strongly correlated CeIn$_3$ compound. To this end, we have considered two points to increase the accuracy of our theoretical predictions: (i) Above the Néel temperature, the 4f-spins and -electrons may lose coherence up to the coherence temperature. This can lead to a different, yet varying electron scattering process. At higher temperatures, the other scattering processes, as indicated in “Calculation details” section, may also occur. There are several approaches to consider these scattering processes. The most systematic approach may be employing temperature dependent DFT. However, in this paper, we have used a simpler but faster approach using the original Kohn-Sham DFT plus semiclassical Boltzmann theory, where temperature is included via the Fermi-Dirac distribution function. By this, we have assumed that the shape of the band structure of the system is not substantially changed and only this is the occupations of the bands that can be changed by the temperature. Although this assumption is reasonable for the temperature range considered here, the presented hydroelectric results can be further improved by applying the temperature dependent DFT approach. It is well-known that at higher temperatures the relaxation time may not be necessarily constant for every system^81^. There are several approaches to go beyond the constant relaxation time assumption. In this paper, we have used an energy dependent models with two different scattering parameters. By this, we have made temperature dependent the relaxation time. Changing the lifetime between mode1 and model2 can change the electrical conductivity. Not all quantities change, but it is evident that assumptions on the temperature-dependent lifetimes and exchange-correlation functional (including +U and DMFT) can affect the computed quantities and figure of merit. Another approach that we have not followed here is fitting the relaxation time to the available experimental data. To this end, one can find the temperature dependent values of $\tau$ for up and down channels so that the calculated thermopower and electrical conductivity are well fitted to the corresponding experimental data. Although the latter may not be considered as an *ab initio* calculation due to the performed matching, the results of this scheme may be closer to the experimental data. (ii) We have quantitatively predicted the dHvA frequencies using different parameters with the anticipation of further experimental investigation to judge which result is much more reliable among them with different parameters used here. (iii) At ambient pressure, a phase transition can occur for CeIn$_3$ from AFM (PM) to PM (AFM) phase in our considered temperature interval [0, 100 K] at the Néel temperature of 10.1 K. In order to consider this phase transition, in addition to the AFM phase, we have set up a supercell to simulate the PM phase with noncollinear spin orientations, as tabulated in Table 4. The DOSs of the AFM phase are not very far from the DOSs of the PM phase, as shown in Fig. 11. This shows that the magnetic ordering may not substantially affect some of the physical properties. This is in agreement with our recent observation that the structural properties of the AFM and FM phase of this compound are very close to each other, see Table 1 of Ref. ^47^. Due to this fact, we have restricted the accuracy of our thermometric results by considering only the AFM phase even for temperatures higher than the Néel temperature. However, we have recently observed that magnetic structures of some 5f-electron compounds can lead to considerable changes in some sensitive physical quantities such as electric field gradient (EFG)^86^. Although thermometric properties may not be so sensitive as EFG, there is still a room for more improving the thermometric properties by using the PM phase at temperatures higher than Néel temperature.

## Conclusion

In this paper, thermoelectric properties are investigated by a combination of density functional and Boltzmann theories for the hole/electron doped CeIn$_3$ compound. The maximum values of the hole-like or positive Seebeck coefficient ($S^{max-pos}$), electron-like or negative Seebeck coefficient ($S^{max-neg}$) electrical conductivity ($\sigma ^{max}$), electronic thermal conductivity ($\kappa _e^{max}$), power factor ($PF^{max}$), and electronic figure of merit ($Z_e^{max}$) are studied versus temperature for temperatures lower than 100 K. The high, intermediate, and low localization degrees of 4f-Ce electrons are considered through the B3PW91 hybrid with $\alpha$ = 0.20, 0.10 and LDA functionals, as well as GGA+U with $\text {U}_{\text {eff}}=5.5~\text {eV}$. To investigate the effects of pressure on the thermoelectric parameters, the calculations are performed at* P* = 0 and 14 GPa. In addition to the constant $\tau$ model, we have considered an energy dependent $\tau$ model with two different scattering parameters, which are called Model1 and Model2. Then, thermoelectric properties are also calculated based on the latter energy dependent models using B3PW91-$\alpha =0.2$ at* P* = 0, as well. Our results reveal a considerable peak in $Z_e^{max}$ at very low temperatures when the 4f-Ce electrons are in the high and intermediate degrees of localization. This peak originates from the high Seebeck coefficient and electrical conductivity (and as a result high power factor) along with low electronic part of thermal conductivity at very low temperatures. This peak is also predicted by Model1 and Model2 for $Z_e^{max}$. Our calculations also show that the $Z_e^{max}$ predicted by Model1 and Model2 is considerably (one order of magnitude) larger than that predicted by the constant $\tau$ model. Based on our results, this peak is constructed by the hole doping. Decreasing the degree of localization pushes this peak to higher temperatures. This occurs due to the deterioration (improvement) of the Seebeck coefficient at low (high) temperatures. In fact, decreasing the degree of localization strongly affects the Seebeck coefficient, but it has not considerable effect on the conductivity parameters. The same result is obtained by imposing pressure. According to these results, it can be concluded that the thermoelectric efficiency of CeIn$_3$ more depends on the Seebeck coefficient than on the conductivity parameters. Decreasing the degree of localization also changes the doping level related to $Z_e^{max}$. Our results also reveal that imposing pressure increases the value of $Z_e^{max}$ peak provided that 4f-Ce electrons lie in the high or intermediate localized regime. However, this is completely reversed at low localized regime; imposing pressure makes worse the $Z_e^{max}$ peak. The effect of pressure on $Z_e^{max}$ and its related doping levels strongly depends on the degree of localization. This is consistent with our recent report on the structural, magnetic, and electronic properties of CeIn$_3$. We have also performed the electronic structure calculations in PM and AFM phases using GGA+U with $\text {U}_{\text {eff}}=5.5~\text {eV}$ at P = 0. For the calculations of the PM phase, we have used the noncollinear version of the WIEN2k code, i.e., the so called WIENncm code. Our results show that the phase transition from AFM to PM phase does not change the DOS of CeIn$_3$ considerably. Hence, it is expected that the thermoelectric parameters of this compound in PM phase may not be very far from the AFM phase. Due to this small difference and in anticipation of further investigations on the thermoelectric of the PM phase, the thermoelectric properties are reported here only for the AFM phase of the CeIn$_3$ compound. We have also predicted the Kondo effect in this compound. To this end, in addition to the DFT calculations, we have performed DFT plus dynamical mean field theory calculations using eDMFT code to predict more accurately the density of sates. Furthermore, we have calculated the phonon dispersion curve of the system by the PHONOPY code. The results show that the system is dynamical stable. Moreover, the phonon calculations predict similar interactions along the symmetrical path of $\Gamma$-M-X-R-$\Gamma$. These calculations also show high degree of localization for the phonon states of the system. In another work, we had shown that 3 bands cross the Fermi level of CeIn$_3$ using B3PW91-$\alpha =0.20$ at* P* = 0 and 14 GPa. In this work, we have calculated the de Haas–van Alphen (dHvA) frequencies for these 3 bands of the system using SKEAF code. We have observed that the dHvA frequency related to one of these bands decreases as pressure increases using B3PW91-$\alpha =0.20$. In agreement with the latter observation, the results show that at ambient pressure this dHvA frequency also decreases as degree of localization decreases. This confirms this experimental observation that the degree of localization reduces as pressure increases.

## References

[1] Grenier C, Georges A, Kollath C (2014). Peltier cooling of fermionic quantum gases. Phys. Rev. Lett..

[2] Zhou C, Birner S, Tang Y, Heinselman K, Grayson M (2013). Driving perpendicular heat flow: ($$p\times n$$)-type transverse thermoelectrics for microscale and cryogenic Peltier cooling. Phys. Rev. Lett..

[3] Chowdhury I (2009). On-chip cooling by superlattice-based thin-film thermoelectrics. Nat. Nanotechnol..

[4] Bell LE (2008). Cooling, heating, generating power, and recovering waste heat with thermoelectric systems. Science.

[5] Snyder GJ, Toberer ES (2008). Complex thermoelectric materials. Nat. Mater..

[6] DiSalvo FJ (1999). Thermoelectric cooling and power generation. Science.

[7] Bian Z, Wang H, Zhou Q, Shakouri A (2007). Maximum cooling temperature and uniform efficiency criterion for inhomogeneous thermoelectric materials. Phys. Rev. B.

[8] Wang, H., Zhou, H., *Exact Solution of a Constrained Optimization Problem in Thermoelectric Cooling*. California Univ Santa Cruz Dept of Applied Mathematics and Statistics (Defense Technical Information Center, 2008). https://books.google.com/books?id=BGv0DAEACAAJ.

[9] Thiébaut E (2017). Maximization of the thermoelectric cooling of a graded Peltier device by analytical heat-equation resolution. Phys. Rev. Appl..

[10] Vandaele K, He B, Van Der Voort P, De Buysser K, Heremans JP (2018). Wet-chemical synthesis of enhanced-thermopower $${\rm Bi}_{1\text{- }x}{\rm Sb}_{x}$$ nanowire composites for solid-state active cooling of electronics. Phys. Rev. Appl..

[11] Yamamoto K, Aharony A, Entin-Wohlman O, Hatano N (2017). Thermoelectricity near Anderson localization transitions. Phys. Rev. B.

[12] Liu D, Zhao F-Y, Yang H-X, Tang G-F (2015). Thermoelectric mini cooler coupled with micro thermosiphon for CPU cooling system. Energy.

[13] Kaushik SC, Hans R, Manikandan S (2016). Theoretical and experimental investigations on solar photovoltaic driven thermoelectric cooler system for cold storage application. Int. J. Environ. Sci. Dev..

[14] Morelli DT, Meisner GP (1995). Low temperature properties of the filled skutterudite CeFe$$_4$$Sb$$_{12}$$. J. Appl. Phys..

[15] Behnia K, Méasson M-A, Kopelevich Y (2007). Nernst effect in semimetals: the effective mass and the figure of merit. Phys. Rev. Lett..

[16] Yamashita T (2014). Colossal thermomagnetic response in the exotic superconductor URu$$_2$$Si$$_2$$. Nat. Phys..

[17] Giazotto F, Heikkilä TT, Luukanen A, Savin AM, Pekola JP (2006). Opportunities for mesoscopics in thermometry and refrigeration: physics and applications. Rev. Mod. Phys..

[18] May AF, McGuire MA, Cantoni C, Sales BC (2012). Physical properties of Ce$${}_{3-x}$$Te$${}_{4}$$ below room temperature. Phys. Rev. B.

[19] Hong S, Ghaemi P, Moore JE, Phillips PW (2013). Tuning thermoelectric power factor by crystal-field and spin-orbit couplings in Kondo-lattice materials. Phys. Rev. B.

[20] Aydemir U (2011). Low-temperature thermoelectric, galvanomagnetic, and thermodynamic properties of the type-i clathrate Ba$${}_{8}$$Au$${}_{x}$$Si$${}_{46-x}$$. Phys. Rev. B.

[21] Zlatić V (2003). Thermoelectric power of cerium and ytterbium intermetallics. Phys. Rev. B.

[22] Wiśniewski P, Zaremba V, Ślebarski A, Kaczorowski D (2015). Electronic properties of CeRh$$_{1-x}$$Ge$$_x$$In; evolution from an intermediate-valence to a localized 4f-state. Intermetallics.

[23] Takaesu Y (2011). Effect of pressure on transport properties of CeIrIn$$_5$$. J. Phys. Conf. Ser..

[24] Pokharel M (2014). Thermoelectric properties of ceal$$_3$$ prepared by hot-press method. Energy Convers. Manag..

[25] Strydom, A., Paschen, S. & Steglich, F. Thermal and electronic transport in the intermediate-valent compound cerhin. *Phys. B Condens. Matter* **378-380**, 793 – 794 (2006). http://www.sciencedirect.com/science/article/pii/S0921452606003723. Proceedings of the International Conference on Strongly Correlated Electron SystemsSCES.

[26] Kaczorowski D, Gofryk K (2006). Thermoelectric power of $${\rm Ce}$$-based intermediate valent systems. Solid State Commun..

[27] Kletowski Z (1989). Thermoelectric power of the $${\rm REIn}_3$$ single crystals where $${\rm RE} = {\rm La}, {\rm Ce}, {\rm Pr}, {\rm Nd}, {\rm Sm}, {\rm Gd}, {\rm Ho}, {\rm ErIn}_3, {\rm TmandLu}$$. Solid State Commun..

[28] Gambino R, Grobman W, Toxen A (1973). Anomalously large thermoelectric cooling figure of merit in the kondo systems $${\rm CePd}_3$$ and $${\rm Celn}_3$$. Appl. Phys. Lett..

[29] Mathur ND (1998). Magnetically mediated superconductivity in heavy fermion compounds. Nature.

[30] Izyumov, A. & Plaksin, G. *Cerium: Molecular Structure, Technological Applications and Health Effects*. Materials Science and Technologies (Nova Science Publisher’s, Incorporated, 2012). https://books.google.com/books?id=QYmAMAEACAAJ.

[31] Knochel, P. & Molander, G. *Comprehensive organic synthesis* (Elsevier Science, 2014). https://books.google.com/books?id=zLpCAgAAQBAJ.

[32] s. Geological Survey, U. *Minerals Yearbook, 2008, V. 1, Metals and Minerals*. Minerals yearbook volume 1: metals and minerals (U.S. Government Printing Office, 2011). https://books.google.com/books?id=tL4hau707bwC.

[33] Keane, C. & Sever, M. *Consumer’s Guide to Minerals:* (American Geosciences Inst, 2013). https://books.google.com/books?id=aeVHAQAAQBAJ.

[34] Kearley, G. & Peterson, V. *Neutron applications in materials for energy*. Neutron scattering applications and techniques (Springer International Publishing, 2015). https://books.google.com/books?id=dD5oBgAAQBAJ.

[35] Bittar EM (2012). Probing the localized to itinerant behavior of the 4f electron in $${\rm CeIn}_{3-x}Sn_x$$ by $${\rm Gd}^{3+}$$ electron spin resonance. Phys. Rev. B.

[36] Berry N, Bittar EM, Capan C, Pagliuso PG, Fisk Z (2010). Magnetic, thermal, and transport properties of Cd-doped $$\rm CeIn_3$$. Phys. Rev. B.

[37] Oomi G, Kagayama T, Sakurai J (1999). High pressure studies of the concentrated kondo compounds $$\text{ Ce }(\text{ In}_{1-x}Sn_x)_3$$. J. Mater. Process. Technol..

[38] Grechnev, G. *et al.* Magnetovolume effect in paramagnetic alloys of $${\rm CeIn}_{3-x}Sn_x$$. *Journal of Magnetism and Magnetic Materials* **157–158**, 677 – 678 (1996). http://www.sciencedirect.com/science/article/pii/0304885395009906. European Magnetic Materials and Applications Conference.

[39] Ebihara T (2008). High pressure electrical resistivity in $${\rm La}$$ doped $${\rm CeIn}_3$$. J. Phys. Conf. Ser..

[40] Elenbaas, R., Schinkel, C. & van Deudekom, C. Heat capacity and electrical resistivity of $${\rm (Ce, La)In}_3$$ and $${\rm Ce(In, Sn)}_3$$. *Journal of Magnetism and Magnetic Materials* **15–18**, Part 2, 979 – 981 (1980). http://www.sciencedirect.com/science/article/pii/0304885380908513.

[41] Benoit A (1980). Magnetic structure of the compound $${\rm CeIn}_3$$. Solid State Commun..

[42] Harrison N (2007). Fermi surface of $${\rm CeIn}_{3}$$ above the néel critical field. Phys. Rev. Lett..

[43] Asadabadi SJ (2007). Electronic structure and electric-field gradient analysis in $$\rm CeIn_3$$. Phys. Rev. B.

[44] Settai R (2006). Change of the fermi surface in $${\rm CeIn}_3$$: From localized to itinerant. Phys. B Condens. Matter.

[45] Settai R (2005). Change of the fermi surface across the critical pressure in $${\rm CeIn}_3$$: the de haas-van alphen study under pressure. J. Phys. Soc. Japan.

[46] Sakai O, Harima H (2012). Band calculations for ce compounds with AuCu_3_-type crystal structure on the basis of dynamical mean field theory: Ii. $${\rm CeIn}_3$$ and $${\rm CeSn}_3$$. J. Phys. Soc. Japan.

[47] Yazdani-Kachoei M, Jalali-Asadabadi S, Ahmad I, Zarringhalam K (2016). Pressure dependency of localization degree in heavy fermion CeIn$$_3$$: a density functional theory analysis. Sci. Rep..

[48] Lawrence JM, Shapiro SM (1980). Magnetic ordering in the presence of fast spin fluctuations: a neutron scattering study of Ce$${\rm In}_{3}$$. Phys. Rev. B.

[49] Sakurai J, Ohyama T, Komura Y (1985). Thermoelectric power and electrical resistivity of Ce(In_1-x_Sn_x_)_3_ and (Ce_1-x_La_x_In_3_. J. Magn. Magn. Mater..

[50] Ilkhani, M., Abolhasani, M. R. & Jalali-Asadabadi, S. Ab initio study of solid CeIn_3_ at high pressures. *Iran. J. Phys. Res.* **8**, 99 (2008). http://ijpr.iut.ac.ir/article-1-273-en.html.

[51] Ilkhani M, Abolhassani MR, Aslaninejad M (2009). First-principles study of the high-pressure suppression of magnetic moments in CeIn_3_. Phys. Rev. B.

[52] Shafiq, M., Ahmad, I. & Jalali-Asadabadi, S. Mechanical properties and variation in soc going from La to Nd in intermetallic RIn$$_3$$ and RSn$$_3$$ (R = La, Ce, Pr, Nd). *RSC Adv.* **5**, 39416–39423. 10.1039/C5RA03597J (2015).

[53] Jalali-Asadabadi S, Cottenier S, Akbarzadeh H, Saki R, Rots M (2002). Valency of rare earths in RIn$$_3$$ and RSn$$_3$$: Ab initio analysis of electric-field gradients. Phys. Rev. B.

[54] Jalali-Asadabadi S, Kheradmand F (2010). Ab initio prediction of magnetically dead layers in freestanding $$\gamma$$-ce(111). J. Appl. Phys..

[55] Kheradmand, F. & Jalali-Asadabadi, S. Structural and electronic properties of cerium from lda+u calculations. *Iran. J. Phys. Res.* **8**, 235 (2008). http://ijpr.iut.ac.ir/article-1-297-en.html.

[56] Kohn W, Sham LJ (1965). Self-consistent equations including exchange and correlation effects. Phys. Rev..

[57] Hohenberg P, Kohn W (1964). Inhomogeneous electron gas. Phys. Rev..

[58] Allen, P. B. *Boltzmann theory and resistivity of metals*, chap. 17, 219–250 (Kluwer, Boston, 1996). https://books.google.com/books?id=o6dzc6hQ41EC.

[59] Ziman, J. *Electrons and Phonons: The Theory of Transport Phenomena in Solids*. International series of monographs on physics (OUP Oxford, 1960). https://books.google.com/books?id=UtEy63pjngsC.

[60] Okuda T, Nakanishi K, Miyasaka S, Tokura Y (2001). Large thermoelectric response of metallic perovskites: $$\rm Sr_{1-x}{\rm La}\rm _{x}{\rm TiO}_{3}$$ (0 <~ x <~ 0.1). Phys. Rev. B.

[61] Madsen GKH, Blaha P, Schwarz K, Sjöstedt E, Nordström L (2001). Efficient linearization of the augmented plane-wave method. Phys. Rev. B.

[62] Blaha P, Schwarz K, Madsen GKH, Kvasnicka D, Luitz J (2001). WIEN2k: An augmented plane waves plus local orbitals program for calculating crystal properties.

[63] Monkhorst HJ, Pack JD (1976). Special points for Brillouin-zone integrations. Phys. Rev. B.

[64] Becke AD (1993). A new mixing of hartree-fock and local density-functional theories. J. Chem. Phys..

[65] Stephens PJ, Devlin FJ, Chabalowski CF, Frisch MJ (1994). Ab initio calculation of vibrational absorption and circular dichroism spectra using density functional force fields. J. Phys. Chem..

[66] Becke AD (1988). Density-functional exchange-energy approximation with correct asymptotic behavior. Phys. Rev. A.

[67] Perdew JP (1992). Atoms, molecules, solids, and surfaces: applications of the generalized gradient approximation for exchange and correlation. Phys. Rev. B.

[68] Madsen GK, Singh DJ (2006). Boltztrap. a code for calculating band-structure dependent quantities. Comput. Phys. Commun..

[69] Yazdani-Kachoei M, Jalali-Asadabadi S (2019). Thermoelectric properties of heavy fermion CeRhIn$$_{5}$$ using density functional theory combined with semiclassical boltzmann theory. RSC Adv..

[70] Xu B, Verstraete MJ (2013). First-principles study of transport properties in os and ossi. Phys. Rev. B.

[71] Laskowski R, Madsen GKH, Blaha P, Schwarz K (2004). Magnetic structure and electric-field gradients of uranium dioxide: An ab initio study. Phys. Rev. B.

[72] Rourke P, Julian S (2012). Numerical extraction of de haas-van alphen frequencies from calculated band energies. Comput. Phys. Commun..

[73] Togo A, Tanaka I (2015). First principles phonon calculations in materials science. Scr. Mater..

[74] Haule K (2015). Exact double counting in combining the dynamical mean field theory and the density functional theory. Phys. Rev. Lett..

[75] Yang J (2008). Evaluation of half-heusler compounds as thermoelectric materials based on the calculated electrical transport properties. Adv. Funct. Mater..

[76] Shekar NC, Rajagopalan M, Meng J, Polvani D, Badding J (2005). Electronic structure and thermoelectric power of cerium compounds at high pressure. J. Alloys Compd..

[77] Vijayakumar V, Vaidya S, Sampathkumaran E, Vijayaraghavan R (1983). High pressure thermopower and electrical resistance measurements in $${\rm CeSn}_3,{\rm CeAl}_3,{\rm CeAl}_2 \text{ and } {\rm CeIn}_3$$. Solid State Commun..

[78] Caffarel M, Giner E, Scemama A, Ramírez-Solís A (2014). Spin density distribution in open-shell transition metal systems: A comparative post-hartree-fock, density functional theory, and quantum monte carlo study of the CuC$$_{2}$$ molecule. J. Chem. Theory Comput..

[79] Tchokonté, M. B. T., Tshabalala, K. G., du Plessis, P. D. V. & Kaczorowski, D. Magnetic substitution in $${\rm CeIn}_3$$. *J. Phys. Chem. Solids* **71**, 181–186 (2010). http://www.sciencedirect.com/science/article/pii/S002236970900314X.

[80] Hegger H (2000). Pressure-induced superconductivity in Quasi-2D CeRhIn$$_{5}$$. Phys. Rev. Lett..

[81] Ashcroft, N. & Mermin, N. *Solid state physics*. HRW international editions (Holt, Rinehart and Winston, Philadelphia, 1976). https://books.google.com/books?id=oXIfAQAAMAAJ.

[82] Kittel, C. *Introduction to solid state physics* (John Wiley and Sons, Inc., New York, 1983), 5th edn.

[83] Tritt TM (2004). Thermal conductivity: theory, properties, and applications.

[84] Kim H-D (1997). Surface and bulk $$4f$$-photoemission spectra of $${\rm CeIn}_3$$ and $${\rm CeSn}_{3}$$. Phys. Rev. B.

[85] Fujita T (1992). Unusual low-temperature properties of ce compounds. J. Magn. Magn. Mater..

[86] Ghasemikhah E, Asadabadi SJ, Ahmad I, Yazdani-Kacoei M (2015). Ab initio studies of electric field gradients and magnetic properties of uranium dipnicties. RSC Adv..

